# Aerobic and anaerobic removal of lead and mercury via calcium carbonate precipitation mediated by statistically optimized nitrate reductases

**DOI:** 10.1038/s41598-020-60951-1

**Published:** 2020-03-04

**Authors:** Marwa Eltarahony, Sahar Zaki, Desouky Abd-El-Haleem

**Affiliations:** Environmental Biotechnology Department, Genetic Engineering and Biotechnology Research Institute (GEBRI), City of Scientific Research and Technological Applications (SRTA-City), 21934 New Borg El-Arab City, Alexandria Egypt

**Keywords:** Microbiology, Environmental sciences

## Abstract

The nonbiodegradability nature of heavy metals renders them resident in food chain and subsequently, destructing the entire ecosystem. Therefore, this study aimed to employ nitrate reduction-driven calcium carbonate precipitation in remediation of lead and mercury aerobically and anaerobically by *Proteus mirabilis* 10B, for the first time. Initially, Plackett-Burman design was employed to screen of 16 independent variables for their significances on periplasmic (NAP) and membrane-bound (NAR) nitrate reductases. The levels for five significant variables and their interaction effects were further optimized by central composite design. The maximum activities of NAP and NAR recorded 2450 and 3050 U/mL by 2-fold enhancement, comparing with non-optimized medium. Under aerobic and anaerobic optimized remediation conditions, the changes in media chemistry revealed positive correlation among bacterial growth, nitrate reductase activity, pH, NO_3_^−^ and NO_2_^−^ consumption and removal of Ca^2+^, Pb^2+^ and Hg^2+^. Subsequently, the remediated precipitates were subjected to mineralogical analysis; energy dispersive X-ray patterns exhibited characteristic peaks of C, O and Ca in addition to Pb and Hg. Scanning electron microscope depicted the presence of bacterial imprints and protrusions on rough and smooth surface bioliths. However, X-ray diffraction indicated entrapment of PbCO_3_, Pb_2_O, CaPbO_3_, Hg and Hg_2_O in calcite lattice. Interestingly, such approach is feasible, efficient, cost-effective and ecofriendly for heavy metals remediation.

## Introduction

Pollution with heavy metals is serious environmental concern, especially with continuous growing in urbanization and rapid pace in population which are synchronized with progressive increase in industrialization. Lead (Pb) and mercury (Hg) are among the “big three” heavy metals, possessing density more than 5 g/cm^3^, which enters environment as industrial effluents^1^. The anthropogenic activities such as electroplating, painting, textile manufacturing, smelting, mine drainage, metallurgy, batteries production, construction, medical treatments, military and coal burning are the major sources of hazardous lead and mercury byproducts^2^. Indeed, the real hazard caused by lead and mercury are assigned to their nonbiodegradability and hence accumulate persistently in the food chain causing severe impact on all living organisms^3^. In general, the neurological disorders, heart disease, permanent brain damage and behavioral abnormalities are the common symptoms of lead toxicity in adults and children. Whereas, Minamata’s disease is one of the most common diseases caused by mercury toxicity^4,5^.

Therefore, there is an urgent need to employ the appropriate method to detoxify these metals from any contaminated wastes or discharges. Recently, conventional remediation techniques were utilized to eliminate them from contaminated environments such as chemical precipitation, evaporation, electrochemical treatment, oxidation/reduction, carbon adsorption, ion exchange, membrane filtration and reverse osmosis^6,7^. Unfortunately, such methods suffer from several limitations such as capital-intensive, less effective and consuming a lot of chemicals and energy as revealed by Bojórquez *et al*.^8^ and Tariq *et al*.^9^. Alternatively, many biological remediation approaches have been introduced proving their efficiency in economic manner as well as environmental friendliness^10^. The bioremediation approach includes the employment of plants, microorganisms, dead biomass or viable cells^11^. However, several studies documented the efficiency of phytoremediation of soil contaminated with different heavy metals, but harsh and dry climate in arid regions limited its applications^12^. Besides, the bioaccumulation, biosorption, bioleaching, biocoagulation and bioflocculation are categorized among biological remediation strategies. The major drawback associated with such methods is the possibility of releasing the adsorbed heavy metals back to the environment^13^. Thus, the biomineralization process considers being an alternative and appropriate method to constrain heavy metals away from the surrounding environment^14^.

Biomineralization or microbially induced carbonate precipitation (MICP) is defined as bioprecipitation of calcium carbonate mineral by the virtue of microbial cells and their biochemical activities. The alkalinity engine and nucleation sites are the fundamental key factors dictated such process^15^. During microbial growth and metabolism activation in MICP process, the microbes are able to generate carbonate compounds (CO_3_^2−^) that react with calcium ions (Ca^2+^) producing CaCO_3_ mineral precipitates^16^. Accordingly, the heavy metals with ionic radius approximate Ca^2+^ such as Pb^2+^ and Hg^2+^ can be incorporated in CaCO_3_ lattice and substituting Ca^2+^ ions. In such way, MICP traps heavy metals, preventing them from liberation in ambient environment^14^. In addition to pollutants bioremediation, several applications were addressed for MICP such as oil recovery, CO_2_ capturing, soil reinforcement, bioplugging/biocementation of rocks, self-healing of concrete structure and restoration of statuary and historical buildings^15,17^.

MICP occurs naturally by diverse microbial species in different environmental conditions through various metabolic pathways, including photosynthesis, oxidative deamination of amino acids, nitrate reduction, urea degradation, sulfate reduction, methane oxidation and extracellular polymeric substances^17,18^. Generally, *Synechococcus* cyanobacteria, *Chlorella sp*.^19^, *Desulfovibrio desulfuricans*^20^, *Bacillus spharicus*^17^, *Sporosarcina sp*.^18^, *Arthrobacter crystallopoietes*, *Rhodococcus qingshengii*, *Psychrobacillus psychrodurans*^21^ and *Verticillium sp*.^22^ are examples on different microbial groups possessing the ability to induce CaCO_3_ precipitation.

It is worth noting that various microbial genera are able to utilize nitrate aerobically and anaerobically by the dint of periplasmic (NAP) and membrane-bound nitrate reductase (NAR), respectively. Thus, they exhibit flexibility in their growth strategy and energy generation; encouraging the exploitation of bacterial nitrate dissimilation in bioremediation of nitrate pollution and detoxification of metals simultaneously in an eco-friendly approach and under different oxygen level. Despite of carbonate yield generated by nitrate dissimilation process is higher than ureolysis (approximately two-fold) and the highly negative standard Gibbs free energy (ΔG° = 785 kJ/mol) provided by it, the published studies about MICP through nitrate dissimilation was limited^23^. As elucidated by Zhu and Dittrich^24^, the published researches about MICP through photosynthesis, ureolysis, nitrate reduction, sulfate reduction, and methane oxidation were 1128, 120, 34, 110, and 44, respectively. Where, the predominant metabolic activity for MICP accounted in terrestrial and aquatic environments (freshwater and marine) were 43% and 38% for ureolysis and photosynthesis, respectively.

Accordingly, this study was conducted to eliminate Pb^2+^ and Hg^2+^ in MICP through aerobic and anaerobic nitrate utilization process. It initiated by maximizing NAP and NAR (the key enzymes for MICP) activities using the optimization statistical approach. Subsequently, under optimized conditions, the precipitation of both metals via MICP process under nitrate dissimilation conditions (aerobically and anaerobically) was assessed and confirmed using energy dispersive X-ray spectrometry (EDS), scanning electron microscopy (SEM) and X-ray diffraction (XRD). In this sense, as far as we know, no study reported before about Pb^2+^ and Hg^2+^ sequestration using MICP through nitrate reduction pathway of *Proteus mirabilis* under aerobic and anaerobic conditions.

## Results and Discussion

### Optimization of NAP and NAR activities by design of experiment (DOE)

#### Evaluation of nutritional/environmental factors affecting NAP and NAR activities using plackett-burman design (PBD)

PBD experiments showed a markedly wide variation in the activities of both enzymes, ranging from 9.14 U/mL to 864.29 U/mL for NAP and 85.71 U/mL to 1322.86 U/mL for NAR along with 20 experimental runs (Tables 1 and 2); this variation reflected the importance of media optimization to attain higher enzyme activity. The multiple linear regression coefficients of the model were analyzed statistically by MINITAB 14 using the student’s t-test. The *p*-values are used to check the consequences and significance of each independent variable within the design (Supplementary Tables S1 and S2). Commonly, the larger magnitude of T-value with ‘a low probability ‘*P*’ value (prob > F < 0.05) indicates high significance of the corresponding coefficient^25,26^.

**Table 1:** Twenty-trial Plackett–Burman matrix for evaluation of independent variables with high/low levels and concentrations along with the actual, predicted NAP activity and studentized residual.

Run Order	X1 pH	X2 Inoculum Size %	X3 FeCl_2_.4H_2_O (mg)	X4 CuSO_4_.5H_2_O (mg)	X5 MnCl_2_·4H_2_O (mg)	X6 Sodium Citrate (g)	X7 K_2_HPO_4_ (g)	X8 MgSO_4_ (mg)	X9 ZnSO_4_·7H_2_O (mg)	X10 CoCl_2_·6H_2_O (mg)	X11 KH_2_PO_4_ (g)	X12 Aeration (rpm)	X13 H_3_BO_4_ (mg)	X14 Na^2^MoO_4_.2 H_2_O (mg)	X15 NaNO_3_ (g)	X16 NaCl (g)	Actual NAP Activity (U/mL)	Predicted NAP Activity (U/mL)	St. Residual
1	6.5	5	0	0	0	1	0	0	0	0	1.5	50	0	0	2	0	117.86	127.06	−0.36
2	6.5	15	400	0	90	5	0	0	0	0	4.5	50	650	0	7	2	371.33	330.93	1.58
3	8.5	15	0	180	90	1	0	0	0	120	1.5	150	0	100	7	2	124.29	94.06	1.18
4	6.5	5	400	0	90	1	3	240	400	120	1.5	50	650	100	2	2	451.43	421.20	1.18
5	6.5	15	400	0	0	1	0	240	0	120	1.5	150	650	100	7	0	684.29	714.52	−1.18
6	8.5	15	400	0	0	5	3	0	400	120	1.5	50	0	0	7	0	186.43	187.40	−0.04
7	8.5	5	0	180	90	1	3	240	0	0	1.5	50	650	0	7	0	9.14	−0.06	0.36
8	6.5	15	400	180	90	1	0	240	400	0	4.5	150	0	0	2	0	263.00	263.97	−0.04
9	6.5	15	0	180	90	5	3	0	0	120	4.5	50	650	100	2	0	287.14	327.54	−1.58
10	6.5	5	400	180	0	5	3	0	0	0	1.5	150	0	100	2	2	864.29	855.09	0.36
11	6.5	15	0	180	0	5	3	240	400	0	1.5	150	650	0	7	2	219.29	228.49	−0.36
12	8.5	5	400	180	0	1	0	0	400	0	4.5	50	650	100	7	2	414.29	454.69	−1.58
13	8.5	15	0	0	0	1	3	0	400	0	4.5	150	650	100	2	0	70.43	30.03	1.58
14	8.5	5	0	0	0	5	0	240	0	120	4.5	150	650	0	2	2	238.57	239.54	−0.04
15	8.5	15	400	180	0	1	3	240	0	120	4.5	50	0	0	2	2	24.53	23.56	0.04
16	6.5	5	0	180	0	5	0	240	400	120	4.5	50	0	100	7	0	797.14	756.74	1.58
17	6.5	5	0	0	90	1	3	0	400	120	4.5	150	0	0	7	2	11.43	41.66	−1.18
18	8.5	5	400	0	90	5	3	240	0	0	4.5	150	0	100	7	0	825.71	834.91	−0.36
19	8.5	5	400	180	90	5	0	0	400	120	1.5	150	650	0	2	0	332.86	331.89	0.04
20	8.5	15	0	0	90	5	0	240	400	0	1.5	50	0	100	2	2	64.29	94.52	−1.18

**Table 2:** Twenty-trial Plackett–Burman matrix for evaluation of independent variables with high/low levels and concentrations along with the actual, predicted NAR activity and studentized residual.

Run Order	X1 KH_2_PO_4_ (g)	X2 MgSO_4_ (mg)	X3 NaCl (g)	X4 CoCl_2_·6H_2_O (mg)	X5 NaNO_3_ (g)	X6 FeCl_2_.4H_2_O (mg)	X7 CuSO_4_.5H_2_O (mg)	X8 K_2_HPO_4_ (g)	X9 pH	X10 ZnSO_4_·7H_2_O (mg)	X11 Na_2_MoO_4_.2H_2_O (mg)	X12 MnCl_2_·4H_2_O (mg)	X13 H_3_BO_4_ (mg)	X14 Sodium citrate (g)	X15 Inoculum Size %	X16 Temp. (°C)	Actual NAR activity (U/mL)	Predicted NAR Activity (U/mL)	St. Residual
1	1.5	0	0	0	3	0	0	0	6.8	0	0	0	0	3	0.5	25	85.71	37.00	1.31
2	1.5	300	1.5	0	7	400	0	0	6.8	0	100	0	630	3	3	35	820.49	854.7	−0.92
3	4.5	300	0	120	7	0	0	0	6.8	400	0	90	0	7	3	35	407.14	430.84	−0.64
4	1.5	0	1.5	0	7	0	180	3	8.3	400	0	0	630	7	0.5	35	205.71	229.41	−0.64
5	1.5	300	1.5	0	3	0	0	3	6.8	400	0	90	630	7	3	25	287.29	263.59	0.64
6	4.5	300	1.5	0	3	400	180	0	8.3	400	0	0	0	3	3	25	461.71	499.91	−1.02
7	4.5	0	0	120	7	0	180	3	6.8	0	0	0	630	3	3	25	189.14	237.85	−1.31
8	1.5	300	1.5	120	7	0	0	3	8.3	0	100	90	0	3	0.5	25	165.71	203.92	−1.02
9	1.5	300	0	120	7	400	180	0	6.8	400	100	0	630	7	0.5	25	808.57	774.36	0.92
10	1.5	0	1.5	120	3	400	180	0	6.8	0	0	90	0	7	0.5	35	673.57	722.28	−1.31
11	1.5	300	0	120	3	400	180	3	8.3	0	0	90	630	3	3	35	324.06	275.35	1.31
12	4.5	0	1.5	120	3	0	0	0	8.3	0	100	0	630	7	3	35	285.71	251.52	0.92
13	4.5	300	0	0	3	0	180	0	8.3	0	100	90	630	7	0.5	25	383.06	417.27	−0.92
14	4.5	0	0	0	3	400	0	3	6.8	400	100	90	630	3	0.5	35	289.71	327.91	−1.02
15	4.5	300	1.5	120	3	0	180	3	6.8	400	100	0	0	3	0.5	35	281.71	243.52	1.02
16	1.5	0	0	120	3	400	0	3	8.3	400	100	0	0	7	3	25	346.07	380.28	−0.92
17	1.5	0	0	0	7	0	180	0	8.3	400	100	90	0	3	3	35	339.01	315.31	0.64
18	4.5	0	1.5	0	7	400	180	3	6.8	0	100	90	0	7	3	25	1322.86	1274.15	1.31
19	4.5	0	1.5	120	7	400	0	0	8.3	400	0	90	630	3	0.5	25	200.00	161.81	1.02
20	4.5	300	0	0	7	400	0	3	8.3	0	0	0	0	7	0.5	35	731.43	707.73	0.64

On the basis of calculated *p*-value, FeCl_2_·4H_2_O (*p*-value, 0.004), Na_2_MoO_4_·2H_2_O (*p* -value, 0.002), sodium citrate (*p* -value, 0.006), inoculum size (%) (*p* -value, 0.009) and pH (*p* -value, 0.009) were considered to be the significant media components that influenced NAP activity, as clearly illustrated in the probability plot of effects and in the Pareto charts (Supplementary Fig. S3-A,B). The probability plot of effects is very important for separating random noise from real effects based on their distribution on the plot. Where, all significant factors that have the largest effect on response lie furthest from the line, while the rest of the factors, which lie along the line, are negligible. Accordingly, FeCl_2_·4H_2_O, Na_2_MoO_4_·2H_2_O, sodium citrate contributed positively to NAP activity (higher concentration of these components was accompanied by increased NAP activity) since these lied on the right-hand side of the line. Whereas, pH and inoculum size (%) negatively affected NAP activity (i.e. increasing in NAP activity was associated with low values of these parameters) as seen as lying on the left-hand side of the line.

On the other hand, FeCl_2_.4H_2_O (*p* -value 0.004), sodium citrate (C-source and electron donor; *p* -value, 0.013), NaNO_3_ (N-source and electron acceptor; *p* -value, 0.026), Na_2_MoO_4_·2H_2_O (*p* -value, 0.042) and pH (*p* -value, 0.028) were the most significant factors affecting NAR activity. As noticed, FeCl_2_·4H_2_O, Na_2_MoO_4_·2H_2_O, NaNO_3_ and sodium citrate positively influenced NAR activity and pH influenced negatively (Supplementary Fig. S4-A). The order of significance was highlighted from a Pareto chart (Supplementary Fig. S4-B).

The standard analysis of variance (ANOVA) indicated that both models were significant in term of small *p* -value (*P* < 0.05), with values of 0.015 and 0.035 obtained for NAP and NAR, respectively (Supplementary Table S5). For examining the overall performance of the model, the coefficient of determination (R^2^) was measured. In addition, the adjusted-R^2^ (adj-R^2^) value should be in reasonable agreement with R^2^ value^27,28^. The model R^2^ and adj- R^2^ values obtained for NAP were 0.9910 and 0.9432, respectively and those obtained for NAR were 0.9837 and 0.8967, respectively. These results imply that 99.10% and 98.37% of the variability of the data can be explained by the model, and there is only a 0.9% and 1.63% chance, which could be due to noise. Both models also exhibited good correlation between the observed (experimental) values and the predicted values, as is evident from the studentized residual (Tables 1 and 2). Smaller residual value is preferred (less than ±2)^29^. Evidently, the residual values of our models fell within this acceptable range.

The first order model for NAP and NAR activities obtained by ANOVA were fitted to the results obtained from the 20 experiments as highlighted in Eqs. 1 and 2:

1 $$\begin{array}{ccc}{\bf{N}}{\bf{A}}{\bf{P}}\,{\bf{A}}{\bf{c}}{\bf{t}}{\bf{i}}{\bf{v}}{\bf{i}}{\bf{t}}{\bf{y}}\,({\bf{U}}/{\bf{m}}{\bf{L}}) & = & 618-88.8\,{\rm{X}}1-11.8{\rm{X}}2+0.620{\rm{X}}3+0.175{\rm{X}}4-\,0.974X5\\  &  & +\,50.4{\rm{X}}6-15.3{\rm{X}}7+332{\rm{X}}8-0.184{\rm{X}}9-0.068{\rm{X}}10+8.31{\rm{X}}11\\  &  & +\,0.911{\rm{X}}12-0.0308{\rm{X}}13+2.81{\rm{X}}14+18.6{\rm{X}}15-39.5{\rm{X}}16\end{array}$$

2 $$\begin{array}{ccc}{\bf{N}}{\bf{A}}{\bf{R}}\,{\bf{a}}{\bf{c}}{\bf{t}}{\bf{i}}{\bf{v}}{\bf{i}}{\bf{t}}{\bf{y}}({\bf{U}}/{\bf{m}}{\bf{L}}) & = & 438+16.5{\rm{X}}1+245{\rm{X}}2+53.4{\rm{X}}3-1.04{\rm{X}}4\\  &  & +\,44.3{\rm{X}}5+0.837{\rm{X}}6+0.761{\rm{X}}7-10.7{\rm{X}}8-115{\rm{X}}9-0.339{\rm{X}}10\\  &  & +\,1.48{\rm{X}}11+0.196{\rm{X}}12-0.162{\rm{X}}13+57.4{\rm{X}}14+24.0{\rm{X}}15+1.08{\rm{X}}16\end{array}$$

For further optimization by the central composite design CCD, all variables with a positive effect on NAP and NAR activities were fixed at a high level, and those variables that had negative effect were maintained at a low level.

#### Central composite design (CCD) for optimization of NAP and NAR activities

To obtain a more precise estimate of the optimal operating conditions, a second order polynomial function was fitted to the experimental results. Thus, the influence of significant variables and interaction effects on the response were investigated. A five-level CCD with 5 independent variables deduced from PBD were applied in a 32-trial matrix. The experimental and the predicted responses along with the design matrix and studentized residual were presented in Table 3.

**Table 3:** Central composite design matrix, representing the response of NAP and NAR activities as influenced by significant factors along with the predicted activity, residuals and concentrations of variables levels.

Run Order	NAP- CCD matrix						Actual NAP Activity (U/mL)	Predicted NAP Activity (U/mL)	St. Residual	Actual NAR Activity (U/mL)	Predicted NAR Activity (U/mL)	St. Residual
	(X1) pH	(X2) FeCl_2_.4H_2_O	(X3) Inoculum Size %		(X4) Na_2_MoO_4_. 2H_2_O	(X5) Sodium citrate						
	NAR- CCD matrix											
	(X1) pH	(X2) FeCl_2_.4H_2_O	(X3) Na_2_MoO_4_. 2H_2_O	(X4) Sodium citrate		(X5) NaNO_3_						
**1**	0	2	0		0	0	1059.29	1063.131	−0.18	1421.0	1396.8	0.65
**2**	−1	1	−1		−1	−1	519.29	537.477	−1.52	819.3	855.8	−1.78
**3**	−1	−1	−1		−1	1	428.57	445.102	−1.39	830.7	830.2	0.02
**4**	1	1	1		−1	−1	1022.14	1022.416	−0.02	748.3	728.5	0.96
**5**	1	1	−1		1	−1	1282.14	1263.797	1.54	386.6	400.4	−0.67
**6**	−1	1	−1		1	1	717.29	714.763	0.21	1666.0	1690.1	−1.17
**7**	0	−2	0		0	0	525	522.478	0.12	1338.0	1393.5	−1.49
**8**	0	0	0		2	0	894.29	929.885	−1.64	276.0	282.4	−0.17
**9**	1	−1	−1		1	1	532.86	512.862	1.68	469.0	445.8	1.13
**10**	0	0	0		0	0	602.86	560.743	1.34	406.7	371.1	0.66
**11**	−1	−1	1		1	1	879.29	879.506	−0.02	1835.2	1810.4	1.21
**12**	0	0	−2		0	0	463.57	466.533	−0.14	558.2	515.9	1.13
**13**	0	0	0		0	0	533.57	560.743	−0.86	306.7	371.1	−1.19
**14**	0	0	0		−2	0	756.43	722.155	1.58	133.2	158.1	−0.67
**15**	−1	−1	1		−1	−1	889.86	910.789	−1.75	1698.8	1686.4	0.6
**16**	1	1	−1		−1	1	1100.29	1096.168	0.35	998.5	990.6	0.38
**17**	0	0	2		0	0	996.57	994.926	0.08	487.1	560.8	−1.97
**18**	−2	0	0		0	0	724.29	686.825	1.73	1595.0	1556.0	1.05
**19**	1	−1	1		1	−1	917.23	901.629	1.31	515.2	480.1	1.71
**20**	−1	1	1		1	−1	1117.29	1119.161	−0.16	594.0	606.1	−0.59
**21**	2	0	0		0	0	1035.43	1074.215	−1.79	393.4	463.7	−1.88
**22**	0	0	0		0	2	739.29	746.441	−0.33	309.3	359.1	−1.34
**23**	−1	−1	−1		1	−1	602.86	605.17	−0.19	387.0	408.3	−1.04
**24**	0	0	0		0	0	558.23	560.743	−0.08	400.0	371.1	0.53
**25**	1	−1	1		−1	1	897.14	895.761	0.12	100.1	43.3	2.77
**26**	0	0	0		0	0	561.6	560.743	0.03	389.9	371.1	0.35
**27**	0	0	0		0	−2	816.43	810.598	0.27	105.0	86.4	0.5
**28**	1	−1	−1		−1	−1	662.86	663.575	−0.06	330.0	319.3	0.52
**29**	0	0	0		0	0	522.14	560.743	−1.23	361.9	371.1	−0.17
**30**	1	1	1		1	1	1334.71	1314.273	1.71	470.0	437.7	1.57
**31**	−1	1	1		−1	1	892.86	908.953	−1.35	337.2	327.6	0.47
**32**	0	0	0		0	0	587.38	560.743	0.85	393.0	371.1	0.4
**Variable**								**Coded levels/Experimental Values**				
								−**2**	−**1**	**0**	**1**	**2**
**pH**								5.8	6.3	6.8	7.3	7.8
**Inoculum Size (0**.**5 McFarland) (%)**								2.5	4	5	7.5	10
**NaNO**_**3**_ **(g/L)**								4	5.5	7	8.5	10
**FeCl**_**2**_.**4H**_**2**_**O (mg/L)**								300	350	400	450	500
**Na**_2_**MoO**_4_.**2H**_**2**_**O (mg/L)**								30	70	100	150	200
**Sodium citrate (g/L)**								4	5.5	7	8.5	10

As observed with the experimental trials, the NAP and NAR activities varied considerably. The lowest NAP activity (428.57 U/mL) was recorded in trail number 3, and the highest activity (1334.71 U/mL) was observed in trail number 30. For NAR, the minimum activity (100 U/mL) was observed in trail number 25, while the maximum activity (1835 U/mL) was achieved in trial number 11 as a factorial point. This variation validates the CCD, in which different concentrations with different interactions result in different response values.

#### Multiple regression analysis and ANOVA

Tests for significance of the regression model, significance of individual model coefficients and lack-of-fit were conducted. The ANOVA results, which are summarized in Tables 4 and 5, demonstrated that both models were highly appropriate and adequate as was evident from model *F*-value (79.0 and 111.69 for NAP and NAR, respectively) with very low probability value (0.000) for both. In addition, the multiple regression analysis explained the role of each individual variable, squared and their second-order interactions on NAP and NAR activities; based on signs (positive or negative effect on the response) and statistical significance of the coefficients (*P* < 0.05). Apparently, the probability values of the coefficients suggested that all linear and quadratic effects of the examined variables were more predominant terms for improving NAP activity than interaction effect. Moreover, the interaction effect of all variables appeared to be significant, with the exception of the interaction between pH & Na_2_MoO_4_.2H_2_O, pH & sodium citrate and Na_2_MoO_4_.2H_2_O & inoculum size (%).

**Table 4:** Estimated effect, regression coefficients and corresponding *T* and *P* values in addition to ANOVA analysis for the optimization of NAP activity using central composite design (CCD).

Term	Coef	SE Coef	T	*P*		
**Constant**	560.743	13.669	41.022	0.000		
**X1**	96.848	6.996	13.844	0.000		
**X2**	135.163	6.996	19.321	0.000		
**X3**	132.098	6.996	18.883	0.000		
**X4**	51.933	6.996	7.424	0.000		
**X5**	−16.039	6.996	−2.293	0.043		
**(X1)**^**2**^	79.944	6.328	12.634	0.000		
**(X2)**^**2**^	58.015	6.328	9.168	0.000		
**(X3)**^**2**^	42.497	6.328	6.716	0.000		
**(X4)**^**2**^	66.319	6.328	10.481	0.000		
**(X5)**^**2**^	54.444	6.328	8.604	0.000		
X1* X2	80.19	8.568	9.359	0.000		
X1* X3	−57.389	8.568	−6.698	0.000		
X1* X4	−12.602	8.568	−1.471	0.169		
**X1 X5**	11.995	8.568	1.4	0.189		
X2* X3	−38.024	8.568	−4.438	0.001		
X2* X4	53.94	8.568	6.296	0.000		
X2* X5	27.453	8.568	3.204	0.008		
X3* X4	7.649	8.568	0.893	0.391		
X3* X5	21.601	8.568	2.521	0.028		
X4* X5	−42.505	8.568	−4.961	0.000		
**Source**	**Df**	**Seq SS**	**Adj SS**	**Adj MS**	***F***	***P***
**Regression**	20	1856884	1856884	92844	79.05	0.000
**Linear**	5	1153267	1153267	230653	196.38	0.000
**Square**	5	424140	424140	84828	72.22	0.000
**Interaction**	10	279478	279478	27948	23.8	0.000
**Residual Error**	11	12920	12920	1175		
**Lack of fit**	6	8201	8201	1367	1.45	0.351
**Pure error**	5	4719	4719	944		
**Total**	31	1869804

**Table 5:** Estimated effect, regression coefficients and corresponding *T* and *P* values in addition to ANOVA analysis for the optimization of NAR activity using central composite design (CCD).

Term	Coef	SE Coef	T	*P*		
**Constant**	371.148	23.5	15.793	0.000		
**X1**	−273.068	12.03	−22.704	0.000		
**X2**	0.828	12.03	0.069	0.946		
**X3**	11.227	12.03	0.933	0.371		
**X4**	31.075	12.03	2.584	0.025		
**X5**	68.174	12.03	5.668	0.000		
**(X1)**^**2**^	159.67	10.88	14.677	0.000		
**(X2)**^**2**^	255.998	10.88	23.532	0.000		
**(X3)**^**2**^	41.795	10.88	3.842	0.003		
**(X4)**^**2**^	−37.722	10.88	−3.467	0.005		
**(X5)**^**2**^	−37.09	10.88	−3.409	0.006		
X1* X2	157.773	14.73	10.711	0.000		
X1* X3	−69.544	14.73	−4.721	0.001		
X1* X4	−70.776	14.73	−4.805	0.001		
X1* X5	−69.541	14.73	−4.721	0.001		
X2* X3	−240.845	14.73	−16.35	0.000		
X2* X4	−2.1	14.73	−0.143	0.889		
X2* X5	38.725	14.73	2.629	0.023		
X3* X4	37.495	14.73	2.545	0.027		
X3* X5	−178.44	14.73	−12.114	0.000		
X4* X5	242.957	14.73	16.494	0.000		
**Source**	**DF**	**Seq SS**	**Adj SS**	**Adj MS**	***F***	***P***
**Regression**	20	7754656	7754656	387733	111.69	0.000
**Linear**	5	1927351	1927351	385470	111.03	0.000
**Square**	5	2765558	2765558	553112	159.32	0.000
**Interaction**	10	3061748	3061748	306175	88.19	0.000
**Residual Error**	11	38188	38188	3472		
**Lack of fit**	6	31186	31186	5198	3.71	0.086
**Pure error**	5	7002	7002	1400		
**Total**	31	7792845				

However, it could be seen from the degree of significance that the linear effects of pH, sodium-citrate and NaNO_3_ and quadratic effects of all variables were significant; meaning that they can act as limiting factors for NAR activity, and little changes in their values will alter the activity rate. Interactions between two factors could be described as an antagonistic effect (negative coefficient) such as the interaction between pH and sodium citrate. While, the interaction between sodium citrate and NaNO_3_ had a synergistic effect (positive coefficient), pointing out that increasing the concentration of both carbon/electron donor source and N/electron acceptor source can lead to enhance NAR activity.

The goodness of fit of the model was checked by calculating the determination coefficient R^2^, which was found to be 0.993 and 0.995 for NAP and NAR respectively, implying that 99.3 and 99.5% of the experimental data of the enzyme activity are compatible with the data predicted by the model, whereas only 0.7% and 0.5% of the total variations are not explained by the model. Besides, the values obtained for adj-R^2^ were 0.981 and 0.986 for NAP and NAR, respectively, indicating good adjustment and reasonable agreement with R^2^ value, thereby confirming high significance and the adequacy of the models.

Furthermore, lack of fit test was also performed. It describes the variation in the data around the fitted model^30^. The insignificant lack-of-fit is desired. It indicates that there might be contributions to the regresses-response relationship that are not accounted for by the model, and hence, a good model will have an insignificant lack-of-fit. The results of the lack of fit test, as inferred by ANOVA (Tables 4 and 5) were insignificant by 0.351 and 0.086 for NAP and NAR, respectively. Generally, results indicated that the models are good and well-fitted to the experimental data. The maximum NAP and NAR activities could be described as a function of the optimum levels of five independent variables. To evaluate the relationship between independent variables and response and to predict the maximum NAP and NAR activities corresponding to the optimum levels of significant variables, a second-order polynomial model (Eqs. 3 and 4) were proposed as follows:

3 $$\begin{array}{ccc}{\bf{N}}{\bf{A}}{\bf{P}}\,{\bf{a}}{\bf{c}}{\bf{t}}{\bf{i}}{\bf{v}}{\bf{i}}{\bf{t}}{\bf{y}}\,({\bf{U}}/{\bf{m}}{\bf{L}})\, & = & 560+96.84{\rm{X}}1+135.16{\rm{X}}2+132.09{\rm{X}}3+\,51.93{\rm{X}}4\\  &  & -\,16.03{\rm{X}}5+79.94{({\rm{X}}1)}^{2}+58.01{({\rm{X}}2)}^{2}+42.49{({\rm{X}}3)}^{2}\\  &  & +\,66.31{({\rm{X}}4)}^{2}+54.44{({\rm{X}}5)}^{2}+80.19{\rm{X}}{1}^{\ast }{\rm{X}}2-57.38{\rm{X}}{1}^{\ast }{\rm{X}}3 \% \\  &  & -\,12.60{\rm{X}}{1}^{\ast }{\rm{X}}4+11.99{\rm{X}}{1}^{\ast }{\rm{X}}5-38.02{\rm{X}}{2}^{\ast }{\rm{X}}3+53.9{\rm{X}}{2}^{\ast }{\rm{X}}4\\  &  & +\,27.45{\rm{X}}{2}^{\ast }{\rm{X}}5+7.64{\rm{X}}{3}^{\ast }{\rm{X}}4+21.60{\rm{X}}{3}^{\ast }{\rm{X}}5-42.50{\rm{X}}{4}^{\ast }{\rm{X}}5\end{array}$$

4 $$\begin{array}{ccc}{\bf{N}}{\bf{A}}{\bf{P}}{\bf{a}}{\bf{c}}{\bf{t}}{\bf{i}}{\bf{v}}{\bf{i}}{\bf{t}}{\bf{y}}({\bf{U}}/{\bf{m}}{\bf{L}}) & = & 371-273{\rm{X}}1+0.828{\rm{X}}2+11.227{\rm{X}}3+31.075{\rm{X}}4\\  &  & -\,68.174{\rm{X}}5+159{({\rm{X}}1)}^{2}+255.9{({\rm{X}}2)}^{2}+41.75{({\rm{X}}3)}^{2}\\  &  & +\,37.722{({\rm{X}}4)}^{2}+37.09{({\rm{X}}5)}^{2}+157.77{\rm{X}}{1}^{\ast }{\rm{X}}2-69.541{\rm{X}}{1}^{\ast }{\rm{X}}3\\  &  & -\,70.776{\rm{X}}{1}^{\ast }{\rm{X}}4+69.541{\rm{X}}{1}^{\ast }{\rm{X}}5-240.845{\rm{X}}{2}^{\ast }{\rm{X}}3+2.1{\rm{X}}{2}^{\ast }{\rm{X}}4\\  &  & +\,38.725{\rm{X}}{2}^{\ast }{\rm{X}}5+37.495{\rm{X}}{3}^{\ast }{\rm{X}}4+178.44{\rm{X}}{3}^{\ast }{\rm{X}}5-242.95{\rm{X}}{4}^{\ast }{\rm{X}}5\end{array}$$

#### Graphical interpretation of the response surface model

**Figure 1:**
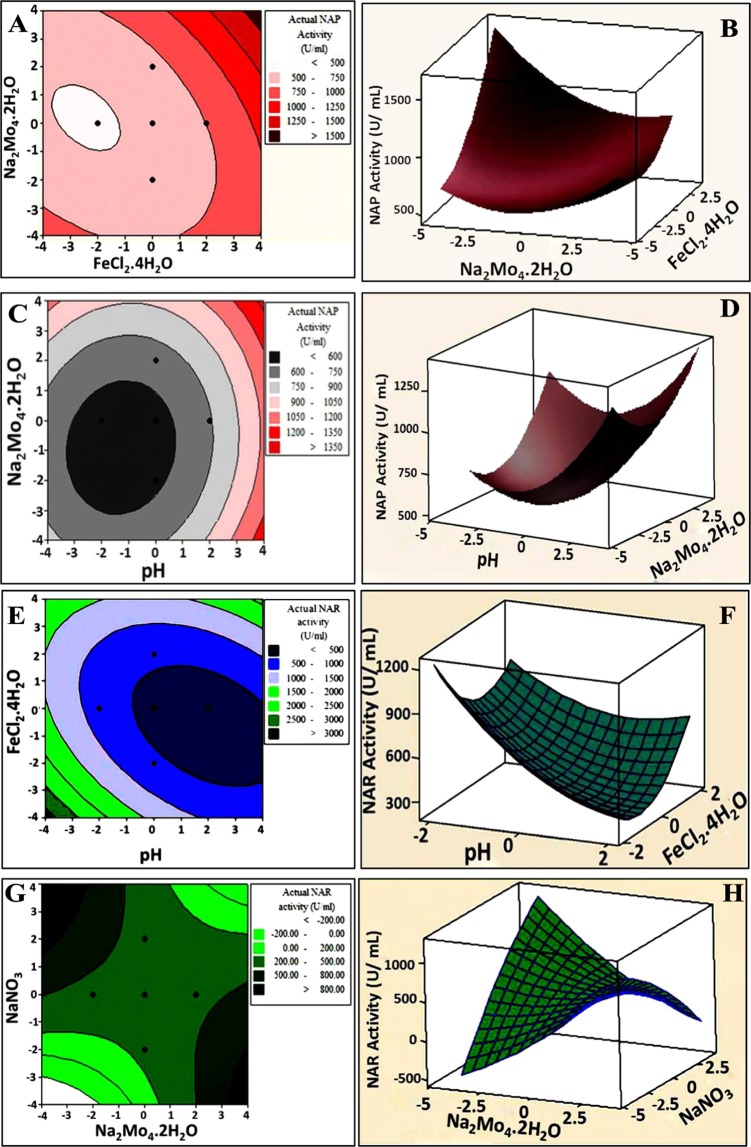
2D-Contour plot (left panels) and 3D-Surface plot (right panels) showing the interactive effects of independent significant variables on NAP activity (**A**–**D**) and NAR activity (**E**–**H**).

The three-dimensional response surface plot and two- dimensional contour plots are graphical representation of the model equations obtained in the regression analysis to figure out the interaction of the studied variables and the optimal levels of each variable predicted for the optimal NAP/NAR activity^31^. The empirical functional relationship is expressed as the response on the vertical axis and coded levels of two explanatory factors on horizontal axes, while the remaining factors are held at the center point (zero levels) (Fig. 1A–H). There was common manner exhibited by surface plots of both NAP and NAR. As noticed, the plot was a U-shaped parabola, which opened upward and had the stationary point directed to be minimum.

**Figure 2:**
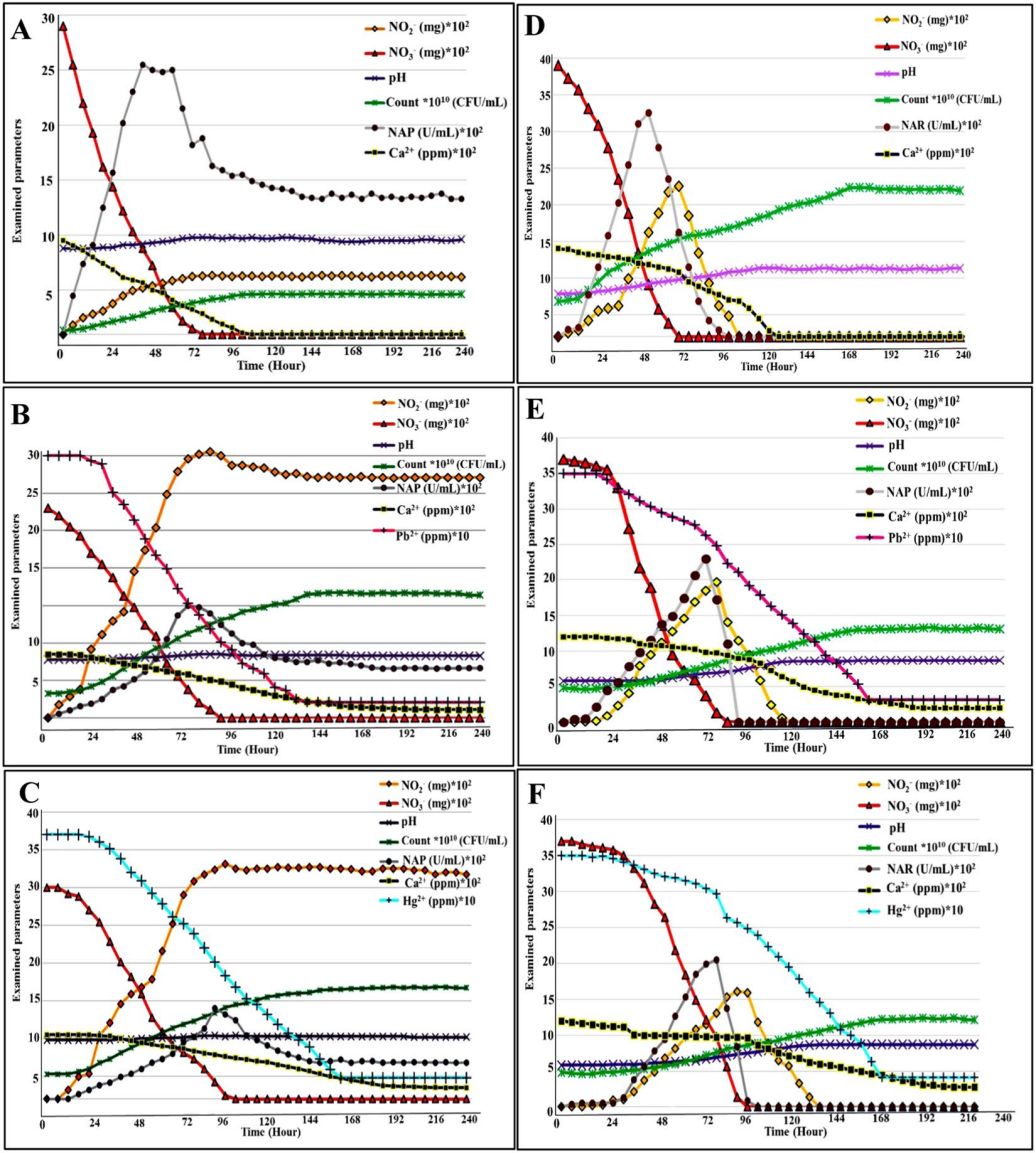
Bioremediation process of Pb^2+^ and Hg^2+^ through aerobic and anaerobic nitrate utilization and study of the changes in media chemistry during MICP. (**A)** Aerobic biotic control; (**B**) Aerobic remediation of Pb^2+^; (**C**) Aerobic remediation of Hg^2+^; (**D**) Anaerobic biotic control; (**E**) Anaerobic remediation of Pb^2+^ and (**F**) Anaerobic remediation of Hg^2+^. The average of three replica were performed for each one. To adjust the scale, the parameters are multiplied in factor as indicated on the figures.

Figure 1(A,B) represents a 2D -contour plot and 3D -surface plot as a function of FeCl_2_·4H_2_O and Na_2_MoO_4_·2H_2_O on NAP activity at a constant values of sodium citrate, pH and inoculum size (%) at their zero levels. It showed that when concentrations of FeCl_2_·4H_2_O and Na_2_MoO_4_·2H_2_O increased, the NAP activity gradually increased. The elliptical contour plot (Fig. 1A) confirmed the significant synergistic interaction between two factors. Broadly, the shape of the contour plot points out the nature and extent of the interactions between the variables. Elliptical and saddle-shaped contour plots elucidate a significant interaction between variables, whereas, a circular contour plot reveals an insignificant interaction between variables^25^. As a consequence, the correlation between Na_2_MoO_4_·2H_2_O and pH was considered to be insignificant (Fig. 2C). Maximum NAP activity could be achieved by increasing the concentration of Na_2_MoO_4_·2H_2_O while decreasing pH value or vice versa, implying the antagonistic interaction (Fig. 2D).

Nonetheless, maximum NAR activity was clearly observed at a low pH value, which was associated with a low concentration of FeCl_2_·4H_2_O, reflecting a synergistic interaction between them (Fig. 1E,F). On the other hand, a ridged surface plot and a saddle-like stationary point was observed for the response, reflecting an antagonistic effect of the interaction between Na_2_MoO_4_·2H_2_O and NaNO_3_ on NAR activity, as shown in Fig. 1G,H. NAR activity increased with increasing Na_2_MoO_4_·2H_2_O and decreasing in NaNO_3_ concentration or vice versa. From the corresponding contour plot, a significant interaction was observed between Na_2_MoO_4_·2H_2_O and NaNO_3_.

The reduced regression model was solved for predicting the maximum NAP/NAR activity using the Response Optimizer tool in MINITAB 14.0. Minitab’s Response Optimizer calculates individual desirability using a desirability function (also called utility transfer function). The predicted optimal levels of the process variables for NAP activity of strain 10B were as follows: pH, 7.8; Na_2_MoO_4_·2H_2_O, 200 mg/L; FeCl_2_·4H_2_O, 500 mg/L; sodium citrate, 4 g/L; and inoculum size, 0.5 McFarland 2.5%. On the other hand, for NAR activity, the following optimal values recorded: pH, 5.8; NaNO_3_, 10 g/L; sodium- citrate, 10 g/L; Na_2_MoO_4_·2H_2_O, 200 mg/L; and FeCl_2_·4H_2_O, 300 mg/L.

#### Experimental verification of model

In order to determine the accuracy of both models and to verify the results, using the optimized conditions suggested by CCD, an experiment was carried out in triplicate and in parallel with basal media before optimization process. The optimization strategy led to a 2.14-fold enhancement of NAP activity from 1161/mL to 2490 U/mL. While, NAR activity was 1522 U/mL under basal conditions, and improved to 3050 U/mL by 2-fold increase under optimized conditions.

It is noteworthy that molybdenum cofactor (Mo-co) and cytochrome subunits (haem or iron–sulphur clusters [4Fe-4S]) are incorporated in the NAP/NAR active center in the catalytic subunit^32^. Moreover, iron has the vital role in iron-rich electron transfer systems (ETS), which are essential for ATP synthesis in aerobic heterotrophic bacteria, as referred by Kirchman *et al*.^33^. This result is in agreement with the finding of Eltarahony *et al*.^34^ who highlighted the importance of molybdenum and iron for NAP of *Achromobacter sp*. MMT. It should be noted that sodium citrate is widely used as a carbon/electron source for anaerobic denitrification process^35,36^. It is clear that electrons generated from carbon substrate metabolism are conserved for NO_3_^−^ reduction and subsequently drive energy generation. As reported by Gamez *et al*.^37^ and Li *et al*.^38^, the metabolic pathway used for citrate utilization under anaerobic conditions varied from that used aerobically (i.e., the TCA cycle). In general, three different fermentation pathways have been described in enterobacteria for anaerobic utilization of citrate. Actually, it is converted to several products, such as acetate, propionate, formate, pyruvate and succinate ended by CO_2_, all of which participate in the electron donating cycle.

In fact, the statistical design was substantially successful at finding an equilibration between cell density (2.5%) and substrate concentration (4 g/L) to maximize NAP activity. In contrast, nitrate reduction by strain *Zoogloea* N299 showed no significant difference among the tested inoculums dosages (2–10%)^36^. Whereas, increasing the initial cell count of *M*. *roseus* and *E*. *coli* O157:H7 elevated nitrate reduction rate after 24 hr. of incubation^39^.

Apparently, NAP and NAR activities of strain 10B seemed to be pH dependent but differently. As reported in several previous studies, the most adequate pH for nitrate reduction always ranged from the neutral to slightly alkaline conditions (6.5–7.8), which is in concordance with our results for NAP enzyme^34,40^. On the other hand, the maximum NAR activity was predicted under initial acidic conditions (5.8). That could be explained by the generation of some byproducts such as oxygen hydroxide (OH^−^) along with carbon dioxide (CO_2_^−^), which were formed from the anaerobic reduction of nitrate concurrently with citrate utilization. The interaction of these products generates bicarbonate HCO_3_^−^ and carbonates CO_3_^2−^ and may induce a certain amount of alkalinity, which donates buffering capacity as suggested by Drtil *et al*.^41^. Consistent with our results, Aoki *et al*.^42^ documented that *Micrococcus denitri*fican and *M*. *halodenitrificans* that were isolated from rice field soil had optimum pH at 5.6 and 6.3, respectively, for nitrate removal. Notably, under aerobic conditions, facultative anaerobes, which are capable of performing denitrification, prefer oxygen to nitrate as electron acceptor as oxygen has a higher redox potential (+818 mV) than nitrate (420 mV)^43^. Hence, nitrate was not a critical parameter to improve NAP activity in this study.

### Bioremediation of Pb^2+^ and Hg^2+^ in MICCP process

The ability of strain 10B to precipitate Pb^2+^ and Hg^2+^ via nitrate dissimilation under oxic/anoxic conditions and the changes in media chemistry were monitored. This was performed through assessment of some parameters as a function of time for experimental trials, biotic (without metals) and abiotic (without bacteria) controls. Broadly, there was a positive correlation between bacterial growth and NAP/NAR activity which were synchronized with pH elevation, removal of soluble (Ca^2+^, Pb^2+^ and Hg^2+^) and NO_3_^−^/NO_2_^−^ reduction as deduced from Fig. (2). In the biotic control, the growth profile of strain 10B exhibited a typical growth stages (lag, logarithmic and stationary) aerobically and anaerobically. As depicts in Fig. 2A, under aerobic conditions, the cell number in biotic control increased rapidly and exhibited maximum NAP activity at 48 hr. by 2450 U/mL with cell density assessed by 17.6 × 10^8^ CFU/mL. Besides, the measured pH increased consistently during incubation from initial pH 7.8 and recorded 8.7 by the end of the experiment. Additionally, a complete NO_3_^−^ reduction was noticed at 84 hr. with accumulation of NO_2_^−^. As initially presented in the optimized MICP media (aerobically), the soluble Ca^2+^ depletion by 98.4% (from 850 ppm to 13.7 ppm) at 108 hr. provided an evidence on CaCO_3_ precipitation.

On the other hand, the overall decrease in cell density and retardation of growth phases were observed in presence of Pb^2+^ and Hg^2+^. That could be possibly assigned to the acclimatization stage of bacteria upon being exposed to stress factor in culture media. In the same extent, Mwandira *et al*.^44^ noticed delay in log phase and noticeable decrease in the growth of *Pararhodobacter sp*. in presence of Pb^2+^. In the present study, the logarithmic phase began after 12 and 18 hr. for Pb^2+^ and Hg^2+^, respectively. The maximum NAP activity displayed by 11.3 × 10^8^ CFU/mL and 11.4 × 10^8^ CFU/mL were 1500 and 1200 U/mL in 78 and 96 hr. for Pb^2+^ and Hg^2+^, respectively (Fig. 2B,C). This implied the effect of both metals on overall bacterial cell performance including enzyme activity^44^. A gradual uplifting in pH was observed from 7.8 to 8.4 for both aerobically remediated metals. Furthermore, nitrate was totally reduced at 96 and 108 hr. for Pb^2+^ and Hg^2+^, correspondingly with accumulation of nitrite as noticed in the biotic control. Remarkably, effective removal percentage reached to 95.2% for Pb^2+^ and 92% for Hg^2+^ were displayed within 144 and 168 hr., respectively as inferred by ICP-OES analysis. Where, the remaining concentration of soluble Pb^2+^ and Hg^2+^ assessed 17 and 28 ppm, from initial concentration of 350 ppm (½ MIC of each heavy metal, data not shown). Further, by 240 hr. of incubation, the measured Ca^2+^ in aqueous media recorded 113 and 151 ppm, from initial concentration of 850 ppm, for Pb^2+^ and Hg^2+^, respectively, which is equivalent to 86.6 and 82.3% of its precipitation.

In comparison, the anaerobic cultures (13.3 × 10^8^, 8.8 × 10^7^ and 8.5 × 10^7^ CFU/mL) of the biotic control, MICP cultures with Pb^2+^ and Hg^2+^ completely reduced NO_3_^−^ by 72, 90 and 108 hr., respectively. The maximum NAR activity evaluated by 3050, 2300 and 2050 U/mL at 54, 78 and 90 hr., for biotic control, Pb^2+^ and Hg^2+^ remediated cultures, respectively (Fig. 2D–F). However, the reduction of whole NO_2_^−^ accomplished in 108, 126 and 150 hr. as the previous order. In accompanying to the denitrification process, the elevation in pH was observed by 9.3 for biotic control and 8.9 for both remediated samples. Such increasing in pH contributed mainly in deposition of 1200, 1008 and 925 ppm of Ca^2+^ in the form of CaCO_3_ crystals with removal percentage evaluated by 100, 84 and 77% for the biotic control, Pb^2+^ and Hg^2+^ remediated cultures, correspondingly. Despite the anoxic precipitation of 91.1% (Pb^2+^) and 88.3% (Hg^2+^), correspond to 319 and 309 ppm by 168 and 186 hr., respectively, it seemed to be slower and with slightly lower performance than aerobic bioremediation. That could be explained by higher redox potential under aerobic conditions as reported previously by Ilbert & Bonnefoy^43^. That led to higher nitrate reduction, bacterial metabolic activity and proliferation, therefore availability of more nucleation sites and eventually faster bioremediation of soluble pollutants. Interestingly, no precipitation was found in the abiotic (chemical) control, reflecting the importance of bacterial activity for altering the physicochemical parameters of the culture media which consequently promoted CaCO_3_ precipitation. It is important to mention that the higher rate of metals removal was obviously occurred during log phase as reported by Kang *et al*.^45^, which concurred with our finding.

Apparently, the nitrate utilization process is the main mechanism that conducted metals precipitation in the current study. Notably, NAP enzyme played a crucial role in rapid utilization of NO_3_^−^ in association to citrate breakdown and protons consumption. Such subsequently led to bicarbonate generation and pH increasing, which ultimately favors CaCO_3_ precipitation. As referred by Singh *et al*.^46^, the increasing in pH and alkalinity as a result of bicarbonate production are coupled with 1 M of NO_3_^−^ reduction and 1 M of proton consumption. The Eqs. (5–7) were suggested to be followed by strain 10B under oxygen availability^47,48^:

5 $$3{{\rm{C}}}_{6}{{\rm{H}}}_{6}{{\rm{O}}}_{7}{{\rm{Na}}}_{2}+16{\rm{Ca}}{({{\rm{NO}}}_{3})}_{2}+16{{\rm{H}}}^{+}\to 16{{\rm{NO}}}_{2}\uparrow +18{{\rm{CO}}}_{2}\uparrow +{{\rm{H}}}_{2}{\rm{O}}$$

6 $${{\rm{CO}}}_{2}+{{\rm{H}}}_{2}{\rm{O}}\leftrightarrow {{{\rm{HCO}}}_{3}}^{-}+{{\rm{H}}}^{+}\ldots \ldots \,\ldots $$

7 $${{{\rm{HCO}}}_{3}}^{-}+{{\rm{Ca}}}^{2+}\leftrightarrow {{\rm{CaCO}}}_{3}+{{\rm{H}}}^{+}\ldots \ldots $$

On the other hand, the following equation could recap the anaerobic denitrification mechanism for CaCO_3_ biodeposition^23,49^

8 $$5{{\rm{C}}}_{6}{{\rm{H}}}_{6}{{\rm{O}}}_{7}{{\rm{Na}}}_{2}+9{\rm{Ca}}{({{\rm{NO}}}_{3})}_{2}\to 9{{\rm{CaCo}}}_{3}\downarrow +9{{\rm{N}}}_{2}\uparrow +21{{\rm{CO}}}_{2}\uparrow +10{{\rm{H}}}_{2}{\rm{O}}+10{\rm{NaOH}}$$

where, strain 10B oxidized the carbon and electron donor (citrate) and reduced nitrate (electron acceptor) by NAR enzyme to drive energy and enhance cells for proliferation. Additionally, higher pH was recorded under oxygen limitation as a consequence of complete reduction of both NO_3_^−^ and NO_2_^−^ and simultaneous utilization of more protons. Singh *et al*.^46^ and O’Donnell *et al*.^47^, stated that the NO_2_^−^ reduction is a decisive step in denitrification process. As more and continuous consumption of H^+^ and higher yield of bicarbonate ions occurred at this stage. Evidently, the variance in physiological functions of both NAP and NAR led to differences in their expression along with MICP process. Where, NAP enzyme was actively expressed till the accomplishment of MICP, even after the complete depletion of NO_3_^−^ and in the accumulation of NO_2_^−^. Where, it conserves redox balance by dissipating excess reductant during aerobic growth and scavenging toxic concentrations of nitrate and nitrite as documented by Li *et al*.^50^. However, NAR enzyme was induced only anaerobically in the presence of NO_3_^−^ and its activity was stalled thereafter. Where, the main function is to produce electrochemical proton gradient and generation of ATP via nitrate respiration^51^. Therefore, nitrate reductases (NAP and NAR) serve as a good bioindicators in the MICP process and subsequently removal of Pb^2+^ and Hg^2+^.

Accordingly, under rising of pH and alkalinity in the culture media, the bio precipitation/crystallization process is commenced via two steps, including crystal nucleation and crystal growth. Actually, the bacterial cells themselves acting as nucleation sites by the virtue of their electronegativity nature. Where, the negative charges biomolecules such as peptidoglycans, teichoic acids, lipids and lipopolysaccharide contain functional groups such as carboxylic (R-COO^−^), sulfonate (R-SO_3_^−^) and phosphatic (R-PO_4_^2−^) attract positively charged ions by attractive van der Waals forces ^52^.Thus, by generation of CO_3_^−^ ions and in the presence of Ca^2+^ ions, CaCo_3_ precipitated and accumulated on the cell surface according to the Eqs. 9–11^53–55^.

9 $${{\rm{Ca}}}^{+2}+{{\rm{cell}}}^{-}\to {\rm{Cell}}-{{\rm{Ca}}}^{+2}$$

10 $${{{\rm{HCO}}}_{3}}^{-}\leftrightarrow {{\rm{H}}}^{+}+{{{\rm{CO}}}_{3}}^{-2}$$

11 $${\rm{Cell}}-{{\rm{Ca}}}^{+2}+{{{\rm{CO}}}_{3}}^{-2}\to {\rm{Cell}}-{{\rm{CaCO}}}_{3}\downarrow $$

**Figure 3:**
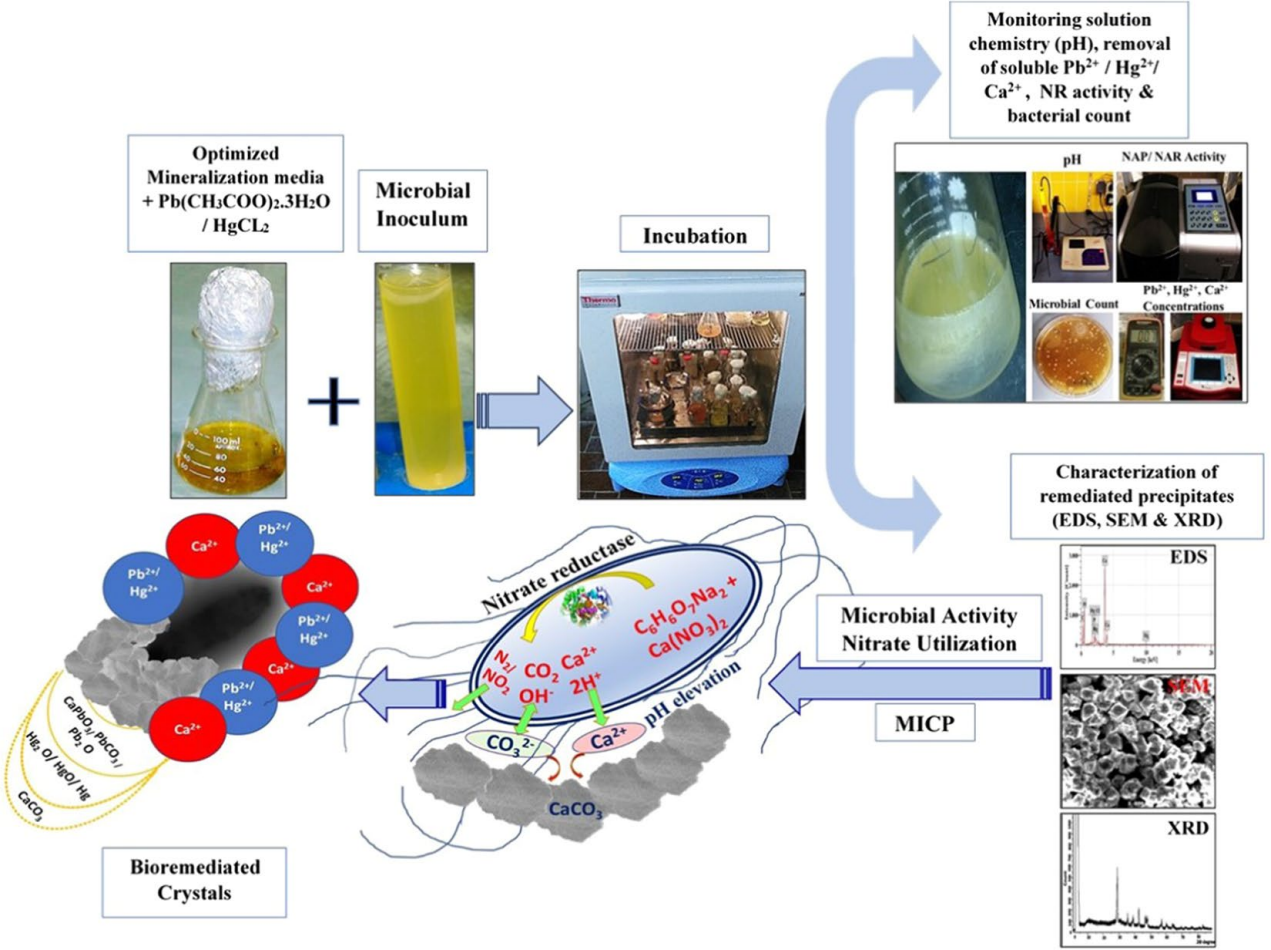
Schematic illustration of Pb^2+^ and Hg^2+^ bioremediation in MICP process through nitrate utilization strategy.

Actually, the ionic selectivity of the cell promotes Ca^2+^ ions to be adsorbed on the cell envelope more frequently to be accumulated inside it. As pointed out by Anbu *et al*.^56^, the cellular ATP-dependent pump, which is located close to outside of the cell, transfers Ca^2+^ ions actively extracellularly; which is coupled with H^+^ uptake. Additionally, Desrosiers *et al*.^57^ and Norris *et al*.^58^ referred that the extracellular concentration of Ca^2+^ ions is up to 103 times higher than intracellular concentration, hence great tendency for extracellular biodeposition. Once the CaCO_3_ nuclei is formed in supersaturated solution, the crystal growth begins through atom-by-atom addition and increase in particles size as described by Trushina *et al*.^59^. Figure 3 illustrated graphical diagram summarizing the whole process.

Regarding the bioremediation of Pb^2+^ and Hg^2+^, ICP-OES results of soluble metals denoted their transformation to insoluble form encapsulating within CaCO_3_ matrix. Such would be approved through the upcoming characterization approaches. The entrapment of both metals via microbially driven biomineralization process alleviated their toxicity through obstruction of their release and decreasing their availability. The incorporation of both metals into CaCO_3_ matrix occurred through substitution of Ca^2+^ with Pb^2+^ and Hg^2+^, especially with presence of unconsumed soluble Ca^2+^ in the range of 11.7–23%, for both metals and under both conditions. This ionic exchange between divalent cations depends on several parameters such as metals hydrolysis constant, electronegativity, ionic radius, hydrated radius and atomic radius as reported by conventional theory^24,60–62^. In the current study, the probability of both metals to be accessed into the interstice of the precipitated crystals and also the similarity in ionic radii among Ca^2+^, Pb^2+^ and Hg^2+^ were suggested. Where, it records 0.1, 0.119 and 0.110 nm for Ca^2+^, Pb^2+^ and Hg^2+^, respectively^63–65^. In coincidence with our results, Chada *et al*.^66^ and Zhu and Dittrich^24^, assigned the existence of divalent cations such as Cd^2+^, Pb^2+^, Sr^2+^, Zn^2+^ and Co^2+^ in calcite crystals to their ionic radii that are closed to Ca^2+^. In the same extent, Mwandira *et al*.^44^ found that the ureolytic strain *Pararhodobacter sp*. detoxified Pb^2+^ completely in MICP process through its incorporation in calcite and vaterite lattice. Whereas, Qian *et al*.^67^ attributed the removal of toxic Cr^6+^ and Pb^2+^ by ureolytic fungi *Penicillium chrysogenum* in MICP process through the steady replacement of CO_3_^2−^ anion in calcite lattice. Despite sufficient removal of Hg^2+^, it required more time for detoxification in comparison to Pb^2+^. That could be ascribed to its more toxicity to the microbial cell due to its potent affinity for thiol (SH) groups of proteins. Therefore, prolonged time was required to be adapted for toxicity^68^. Additionally, microorganisms exhibit preference property for some certain metals over the others as elucidated by Halttunen *et al*.^69^.

### Mineralogical analysis and biodeposits characterization

The obvious and full view of bioremediation efficiency based on MICP of strain 10B under aerobic and anaerobic nitrate reduction conditions would be achieved through EDS, XRD and SEM analysis. These analytical techniques were employed to characterize the precipitated crystals (biotic controls and remediated samples) as follows:

#### EDS

**Figure 4:**
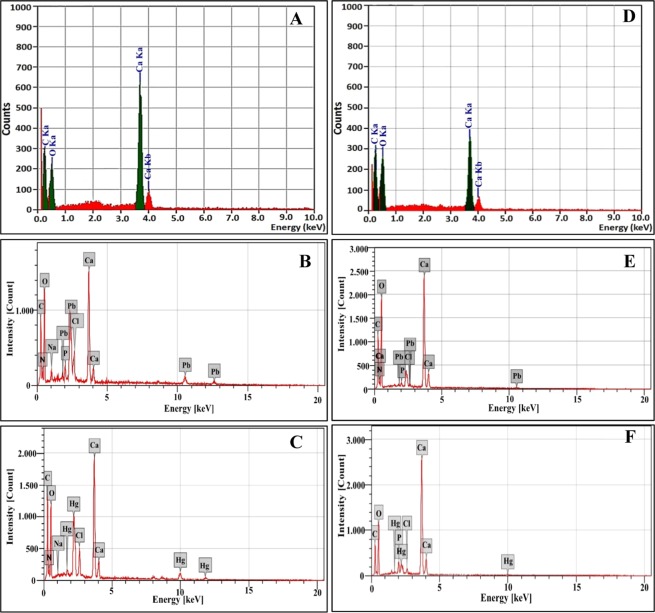
EDS profile of precipitated crystals biotic controls and remediated samples by strain 10B through aerobic and anaerobic nitrate reduction. (**A**) aerobic biotic control; (**B**) Pb^2+^ remediated crystals under aerobic conditions; (**C**) Hg^2+^ remediated crystals under aerobic conditions; (**D**) Anaerobic biotic control; (**E**) Pb^2+^ remediated crystals under anaerobic conditions and (**F**) Hg^2+^ remediated crystals under anaerobic conditions.

EDS microanalysis of crystals precipitated aerobically and anaerobically in biotic controls illustrated in Fig. 4A–D, respectively. Both spectra exhibited characteristic peaks at 0.277, 0.525 and 3.69 keV, which correspond the binding energies of C, O and Ca, respectively^70,71^. Additional peaks related to the binding energy of Pb were detected at 2.293 keV with atomic percentage of 15.2 and 11.7% in aerobic and anaerobic remediated samples, respectively (Fig. 4B–E). EDS also showed the presence of Hg through the characteristic Mα and Mβ emission peaks at 2.19 and 2.28 keV, respectively, along with Lα and Lβ peaks at 9.99 and 11.82 keV, respectively as demonstrated in Fig. 4C–F. Such results confirmed the involvement of Pb and Hg in calcareous remediated precipitations. Remarkably, the percentages of bioprecipitated Pb and Hg under oxic conditions were higher than anoxic one. Moreover, the remediated mercury percentages under both conditions were lower than lead, which agreed with ICP-OES results of remediated supernatants. Clearly, the presence of N and P peaks in considerable percentage reflected the biotic origin of the precipitates. In fact, such elements are intrinsic constituents of microbial cells biomolecules which comprise proteins, nucleic acids, phospholipids and lipopolysaccharides^72^. Besides, other peaks could be detected in a small percentage such as Na and Cl which considered being remaining of media components that integrated during MICP process. This result is in agreement with Han *et al*.^73^.

#### SEM

**Figure 5:**
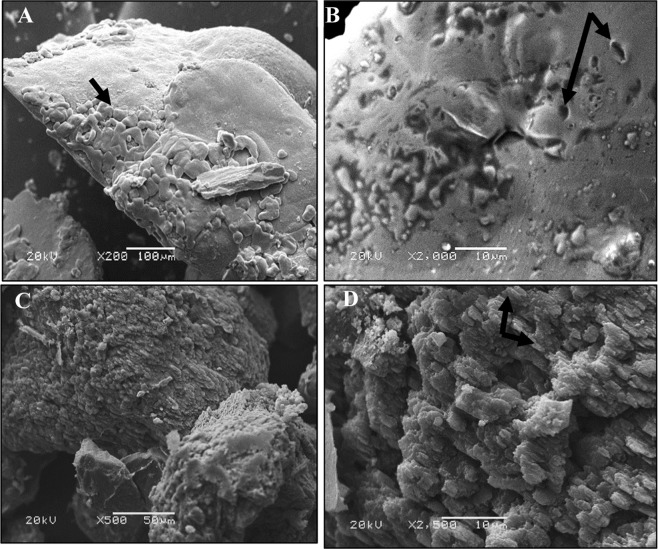
SEM micrographs of Carbonate crystals formed by biotic controls aerobically and anaerobically. (**A**,**B**) Aerobic culture; (**C**,**D**) Anaerobic culture. The arrows referred to the bacterial imprints and protrusions.

The detailed description of biotic controls aerobically and anaerobically was illustrated in Fig. 5. The aerobically mineralized crystals exhibited a fine smooth texture encompassing bacterial cells that were protruded or imprinted above the surfaces as denoted by arrows in Fig. 5A,B. On the other hand, large, rough and coarse particles with wrinkled surface were displayed under anaerobic conditions (Fig. 5C,D). Upon magnification, the calcified cells were assembled compactly into close-packed stacks like superstructures with size ranged from 2 to 5 μm. Similarly, Seifan *et al*.^74^ found differences in the shape and size of CaCO_3_ crystals sedimented by *B*. *licheniformis* ATCC 9789 and *B*. *sphaericus* NZRM 4381 throughout changing incubation conditions such as aeration and pH.

**Figure 6:**
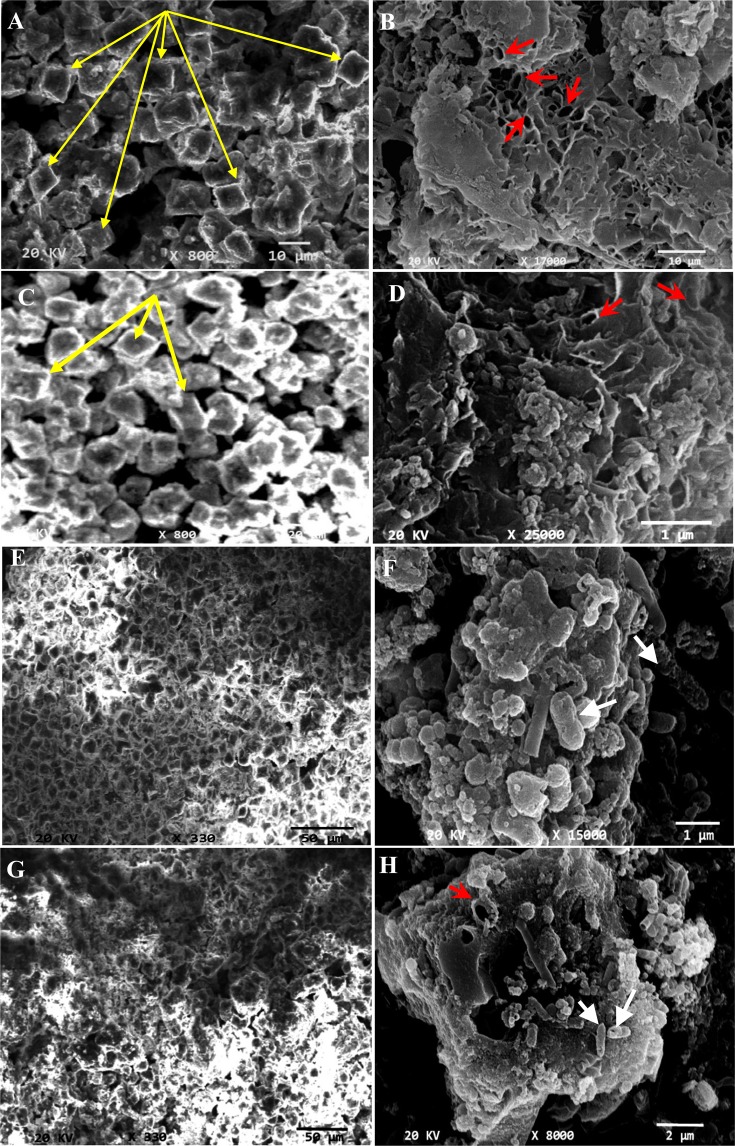
SEM micrographs of remediated crystals precipitated by strain 10B through nitrate reduction process. (**A**,**B**) Pb^2+^ remediated crystals under aerobic conditions; (**C**,**D**) Pb^2+^ remediated crystals under anaerobic conditions; (**E**,**F**) Hg^2+^ remediated crystals under aerobic conditions; (**G**,**H**) Hg^2+^ remediated crystals under anaerobic conditions. The long yellow arrows referred to crystals shape; head white arrows referred to calcified bacterial cells; stealth red arrows referred to the bacterial imprint.

However, some morphological and textural variations were observed in the bioremediated samples. The Pb^2+^ remediated grains showed well-defined faces crystal with rhombic, square as well as cubic shapes as pointed out by yellow arrows (Fig. 6A–C). Besides, the bioremediated crystals generated aerobically showed smooth edges with more angularity than those formed anaerobically. The close view image of aerobic remediated Pb^2+^ (Fig. 6B) displayed layer-flake structures contained sponge like area with many holes. Such holes resembled bacterial cell shape which revealed the delimiting the cell contours by availability of precipitated minerals. Analogues results have been obtained by Rothenstein *et al*.^75^ and Silva-Castro *et al*.^76^. Further, magnified view of the anaerobically remediated Pb^2+^ depicted irregular flaks like matrix embedded with accumulation of calcified cells (Fig. 6D). Whereas, less crystallinity and amorphous shape of Hg^2+^ remediated bioliths were noticed, especially those deposited anaerobically (Fig. 6E–G). The magnified fields of both exhibited rough surface with no angularity as well. The calcified cells were clearly evident on the irregular surface of bioliths as referred by white head arrows (Fig. 6F–H). Moreover, many cavities were displayed on the surface of precipitates (Fig. 6F–H, red stealth arrows), reflecting bacterial imprints, which suggested to be the starting point for the aggregation of carbonate deposits. Commonly, at this stage, the calcified cells entered into death phase due to inhibition of nutrients exchange with the surrounding environment as stated by Silva-Castro *et al*.^76^.

#### XRD

**Figure 7:**
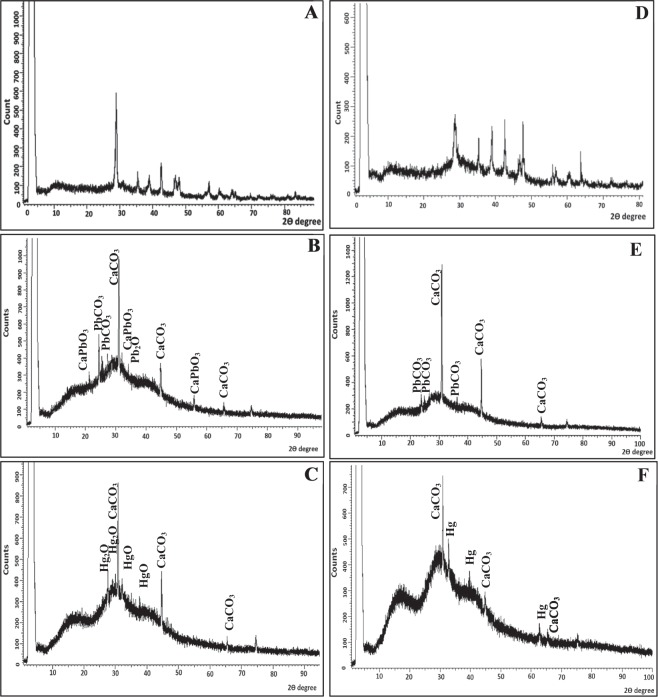
XRD pattern of precipitated crystals biotic controls and remediated samples by strain 10B through aerobic and anaerobic nitrate reduction. (**A**) Aerobic biotic control; (**B**) Pb^2+^ remediated crystals under aerobic conditions; (**C**) Hg^2+^ remediated crystals under aerobic conditions; (**D**) Anaerobic biotic control; (**E**) Pb^2+^ remediated crystals under anaerobic conditions and (**F**) Hg^2+^ remediated crystals under anaerobic conditions.

As depicted in Fig. 7, XRD spectra affirmed the heterotrophic precipitation of CaCO_3._ In the biotic controls under both incubation conditions, sharp, characteristic, distinguishable and broad diffraction peaks of calcite were identified at 2θ values of 29.50, 36.04, 39.51, 43.31, 47.51, 48.65, 56.71, 60.81 and 63.22 that were corresponding to Miller indices of (104) (110), (113), (202), (024), (116), (211), (214) and (125), respectively^77,78^ (Fig. 7A–D). These peaks corroborate with the standard ICDD PDF 5-0586 as referred by Tas,^79^. Generally, XRD crystallographic patterns of all bioremediated samples declared that calcite was the dominant component, which matched with EDS results. The bacterial cells mineralized with calcite together with the remediated metals have been previously mentioned to be common biomineralization products^61,67^. In addition, the diffraction peaks of calcite, especially at 2θ values of 29.50 were shifted to right as a consequence of the replacement or integration of remediated metals in calcite matrix. Reinforces this finding reported by Han *et al*.^80^, who found that Mg^2+^ deteriorated the crystal growth of calcite which thereby lead to right shift in the (104) diffraction peak.

In both Pb^2+^ -remediated samples, the identified phase of Pb^2+^ was cerussite (PbCO_3_); implying simultaneous deposition of Pb^2+^ with CO_3_^2−^ during calcite precipitation induced by nitrate reduction process (Fig. 7B–E). Furthermore, other weak peaks correspond to other phases such as lead oxide (Pb_2_O) and calcium lead oxide (CaPbO_3_) were detected as a product of the bacterial mineralization in the aerobically remediated sample (Fig. 7B). The presence of such phases could be attributed to the uncontrolled metabolic activity that aerobically generated different oxide anions that substituted CO_3_^2−^ group. This result was consistent with the Achal *et al*.^12^, who stated that *Kocuria flava* detoxified Pb^2+^ stress in the contaminated soil through ureolysis process and assigned the formation of different Pb^2+^ phases (carbonate and oxides) to the replacement of CO_3_^2−^ group with Pb oxyanions. Moreover, Achal *et al*.^81^ reported the substitution of As (III) oxyanion for CO_3_^2−^ group via ureolytic *Sporosarcina ginsengisoli* CR5 in calcite.

Regarding Hg^2+^-MICP samples, XRD spectra revealed strong calcite peaks along with weak peaks hardly detected due to background noise; arising from bacterial biomass (Fig. 7C–F). These weak diffraction peaks match those of the standard spectrum PDF 74-0039, PDF 01-0896 and PDF 73-2218, which were assigned to Hg, HgO and Hg_2_O, respectively. In spite of the clear precipitation of HgCO_3_ was not obtained, the remediation process achieved within stable solid phase. Such result could be explained by the flexibility of biogenic calcite structure, including sufficient porosity and surface area, which promoted the incorporation of Hg^2+^ into calcite lattice as documented by Achal *et al*.^72^ and Qian *et al*.^67^. The electronegativity of biogenic calcite suggested favoring the gradual capturing of Hg^2+^ during the crystal growth stage of calcite precipitates. Accordingly, in this case, the produced calcite was acting efficiently not only in the absorption, but also sequestering the toxic metal in stable precipitate. Similarly, Sun *et al*.^82^ recorded such phenomena in removal of Cr(VI) by chemical precipitation. Furthermore, durable encapsulation of Zn^2+^ ions in Ca-Zn-CO_3_ solid solution was obtained by concurrent CaCO_3_ formation and Zn^2+^ sequestration of *S*. *pasteurii* within 7 days remediation process^61^.

Virtually, mineralogical characterization techniques confirmed the presence of Pb^2+^/Hg^2+^-calcite coprecipitated products in the examined remediated sample as possible indirect action of nitrate reductases. In addition, they gave insight on scavenging role of calcite in chelating active soluble heavy metals. Meanwhile, the adsorbed metals were stabilized in calcite matrix and coprecipitated by additional calcite particles which generated continuously during MICP process. Such mixed metal inclusions donated a more potent trap than adsorption which occurs mainly superficially and relies on bonding specifications between adsorbents and adsorbate^83^. It is noteworthy to mention that CaCO_3_ possesses adsorptive capacity and has the ability to retain divalent metals as recorded by Chada *et al*.^66^. That makes it a good ecofriendly agent to apply practically in the uptake of toxic metals arose from anthropogenic activities.

Recently, hydroxide precipitation approach has been utilized effectively in removal of heavy metals^84^. Nonetheless, hydroxide precipitates suffering from redissolution in water through formation of soluble anionic hydroxyl complexes^85^. Hence, the immobilization of toxic metals in carbonate structure via MICP process deemed as alternative approach to alleviate active metals toxicity through limiting their mobility and bio-availability, fix them in stabilized solid and subsequently obstructed their release from calcite lattice to the surrounding ecosystem^61^. In tandem with the current results, Qian *et al*.^67^ manifested that the enhancement of heavy metals removal was conjugated with carbonate precipitations. Interestingly, Sdiri and Higashi,^60^ declared the promising utilization of natural limestones in removal of 10 mg/L of Pb^2+^ in 6 hrs. In this sense, Hu *et al*.^86^ utilized CaCO_3_ as potent alternative to Ca(OH)_2_ to remediate Cu^2+^ with 99.7% removal efficiency. Moreover, Fazlollahi *et al*.^87^ showed that CaCO_3_ coprecipitation technique reduced mercury content in contaminated brine from 0.5 to 0.055 ppm by 89% elimination efficacy.

It is remarkable to mention that Qian *et al*.^67^ found that *Penicillium chrysogenum* CS1 removed 98.8% of Pb^2+^ from initial concentration 200 mg within 12 days. Kang *et al*.^45^ stated that ureolytic strains *Enterobacter cloacae* KJ-46 and KJ-47 removed 60% of Pb^2+^ from initial concentration of 7.2 and 5.9 mg/L, respectively within 48 hr. incubation. Further, François *et al*.^71^ documented that *Kocuria rosea* EP1, *Ochrobactrum* sp. HG16 and *Bacillus* sp. CM111 remediated 100 µM of mercury through biosorption. Moreover, *Pseudomonas putida* succeeded in removal of 4 ppm of Hg^2+^ in two days incubation through volatilization mechanism^88^. Accordingly, strain 10B seemed to be characteristic and considered as a promising tool to remediate high concentrations of Ca^2+^, Pb^2+^ and Hg^2+^under different aeration conditions and in appropriate frame of time. In addition, the higher stability of biogenic carbonate minerals promotes its recruitment *in situ* bioremediation of different contaminated environmental systems such as soil, water and wastewater. Where, its precipitated nature without dispersed pattern facilitates its separation without the requirement to additional step such as filtration, coagulation or flocculation. Finally, the remediation of heavy metals through MICP seems to be advantageous, eco-friendly, sustainable approach and potent alternative to the chemical process.

## Materials and Methods

### Microorganism, cultural conditions and enzyme assay

The bacterial strain *Proteus mirabilis* 10B is a local isolate formerly identified and published as a nitrate reducer and metals nanobiofactory^89^. Both NAP and NAR genes were detected, identified and submitted to the Genbank as described in details elsewhere^72^. The strain was stored in glycerol (20%, v/v) at −20 °C for the forthcoming investigations. The basal medium (g/L) of the following ingredients was used for the optimization process of NAP and NAR enzymes under aerobic and anaerobic conditions: NaNO_3_ 5.0, K_2_HPO_4_·7H_2_O 1.0, KH_2_PO_4_ 3.0, MgSO_4_·7H_2_O 0.12, disodium citrate (C_6_H_6_O_7_Na_2_) 7.5, NaCl 0.5, ZnSO_4_ 0.31, MnCl_2_·4H_2_O 0.03, FeCL_2_·4H_2_O 0.24, Na_2_MoO_4_.2H_2_O 0.015, CuSO_4_·5H_2_O 0.06, CoCl_2_·6H_2_O 0.04 and H_3_BO_3_ 0.057 at pH 7.0 ± 0.2. About 0.5 McFarland equivalents to about 1.5 × 10^8^ CFU/ml was inoculated in 250 mL Erlenmeyer flasks containing 100 mL of the medium and incubated at 30 °C in an orbital shaker (STUART SI500) at 150 rpm (for NAP) and anaerobically (for NAR) as described by Jun^90^ and Siddiqui *et al*.^91^. After the incubation period, the cells were centrifuged at 10.000 X*g* for 20 min at 4 °C and the harvested cells were used to determine NAP and NAR activities according to the procedure followed by Zaki *et al*.^72^. One unit of NAP/NAR activity was identified as the amount of enzyme that catalyzes the formation of 1μmol of nitrite per minute or 1 μmol of nitrate reduced per minute under standard assay conditions.

### Optimization of NAP and NAR activities by design of experiment (DOE)

#### Screening for significant factors influence the NAP and NAR activities by Plackett–Burman Design (PBD)

PBD is a two-level factorial design that is dedicated to screen and identify the controlled experimental factors (nutritional, environmental and incubation conditions) based on their main effect on elevating NAP and NAR activities. PBD allows the investigation of (*n* − 1) variables with at least *n* experiments. Each variable is represented at two levels, high (+) and low (−). Herein, a total of 16 (*n*) variables with two-level concentrations were studied in twenty experiments matrix, as indicated in Tables (1 and 2). Each experiment was performed in triplicate and an average of NAP and NAR activities were calculated as response^25^. Plackett–Burman experimental design is based on the first order model (Eq. 12):

12 $${\rm{Y}}={{\rm{\beta }}}_{O}+\Sigma {\rm{\beta }}{\rm{i}}{\rm{{\rm X}}}{\rm{i}}$$

where, Y is the response or dependent variable (NAP/NAR activity); it will always be the variable we aim to predict, β_o_ is the model intercept and βi is the linear coefficient, and Xi is the level of the independent variable. From the statistical analysis, the main effect was used to elucidate the significance of variables depending on their nature, i.e., positive or negative effect on the production process.

#### Central composite design (CCD) method

This stage was devoted to analyze the interaction among significant variables and also determine their optimal levels. In this study, five different significant variables identified from PBD to greatly influence NAP and NAR activities and were investigated at 5 experimental levels: −*α*, −1, 0, +1, and +*α*. The concentrations of the screened variable at each level in the 32-trail matrix are listed in Table 3. All of the experiments were conducted in triplicate, and the average enzyme activity obtained was taken as the dependent variable or response (Y)^26^. For statistical calculation, the relationship between the coded and actual values is described by Eq. 13:

13 $${\rm{Xi}}={\rm{Ui}}-{{\rm{Ui}}}_{0}/\Delta {\rm{Ui}}$$

where *Xi* is the coded value of the *i*th variable, *Ui* is the actual value of the *i*th variable, Ui_0_ is the actual value of the *i*th variable at the center point and ΔUi is the step change of variable. The second order polynomial structured represented in Eq. 14:

14 $$Y={\beta }_{0}+{\beta }_{1}{X}_{1}+{\beta }_{2}{X}_{2}+{\beta }_{3}{X}_{3}+{\beta }_{11}{X}_{11}+{\beta }_{22}{X}_{22}+{\beta }_{33}{X}_{33}+{\beta }_{12}{X}_{1}{X}_{2}+{\beta }_{13}{X}_{1}{X}_{3}+{\beta }_{23}{X}_{2}{X}_{3}$$

where: *Y* is the predicted response; X_1_, X_2_, X_3_ are input variables which influence the response variable Y*; β*_0_, intercept; *β*_1_, β_2_ and *β*_3_ linear coefficients; *β*_11_, *β*_22_ and *β*_33_, squared or quadratic coefficients *β*_12_, β_13_, and *β*_23_ interaction coefficients

#### Statistical analysis

The statistical software Minitab 14.0 (Minitab Inc., Pennsylvania, USA) was used to perform the experimental designs “trials matrices” and statistical analysis of PDB and CCD. Besides, three-dimensional surface plots and two-dimensional contour plots were constructed to illustrate the relationship between the responses and the different levels of each variables utilized in this study. In addition, the optimizer tool was used to predict the optimum level of experimental factors to maximize response^92^.

#### Validation of experimental model

The statistical model was validated by measuring NAP and NAR activities under conditions predicted by the model and comparing the values to those obtained with basal media^26^.

### Bioremediation of Pb^2+^ and Hg^2+^ in MICP process

The performance of strain 10B in Pb^2+^ and Hg^2+^ bioremediation through aerobic/anaerobic nitrate reduction was examined and confirmed in broth state at flask level. The biomineralization media (the optimized media contained Ca (NO_3_)_2_ instead of NaNO_3_) was supplemented with 1.7 mM (350 ppm) of Pb (CH_3_COO)_2_ .3H_2_O and HgCL_2_. Such concentration was selected according to minimal inhibitory concentration (MIC) test (data not shown). The flasks were inoculated and incubated under aerobic and anaerobic conditions at 30 °C for 10 days. Two controls were run in parallel; the abiotic or negative control contains uninoculated medium and biotic controls containing inoculated biomineralization media without Pb (CH_3_COO)_2_ .3H_2_O or HgCL_2_. The dynamics of bioremediation was evaluated during the biomineralization process, at a constant interval time (6 hr.) through assessment of some parameters, including bacterial count, NAP/NAR activity, pH, concentrations of soluble Ca^2+^, Pb^2+^, Hg^2+^, NO_3_^−^ and NO_2_^−^. A pH meter (PB-10, Sartorius AG) was used to measure the pH; however, NO_3_^−^ and NO_2_^−^ were estimated according to the methods described by APHA^93^. The concentration of soluble Ca^2+^, Pb^2+^, Hg^2+^ were determined by inductively coupled plasma optical emission spectrometer (Agilent ICP-OES 5110DVD) (Central Lab, Alexandria university). Whereas, NAP/NAR activities were determined as described previously; the bacterial count was evaluated by pour plate method. All experiments were conducted in triplicate and the mean values were considered. At the end of the incubation period, all precipitates were centrifuged at 10.000 X*g* for 20 min and subjected to mineralogical analysis^14^.

### Mineralogical analysis and biodeposits characterization

For assessment the identity, morphology, microstructure and chemical constituents of precipitated samples, XRD, SEM and EDS were utilized. XRD was carried out to identify the precipitated minerals by Bruker MeaSrv (D2-208219) diffractometer (Central lab, Faculty of Science, Alexandria University) with Cu Kα tube anode, applying 30KV/30 mA. Scans were run from 0° to 100° 2θ at a scanning speed of 2°/min. However, SEM (JEOL JSM 6360LA, Japan – Advanced Technologies and New Materials Research Institute (ATNMRI), SRTA-City) was used to visualize the morphology of crystals. The elemental composition of the crystals was analyzed with energy dispersive spectrometer SEM (JEOL JSM 6360LA, Japan–Advanced Technologies and New Materials Research Institute (ATNMRI) SRTA-City and JSM-5300, JEOL Japan, Electron microscope unit-Alexandria University) at an operating voltage of 20 kV^94^.

## Conclusion

In conclusion, to the best of our knowledge, this is the first report of an attempt to successfully remediate Pb^2+^ and Hg^2+^ via CaCO_3_ precipitation induced by *Proteus mirabilis* 10B under aerobic and anaerobic nitrate utilization. Therefore, the current study focused on optimization of nitrate reductases aerobically and anaerobically. On the optimized precipitating media, the effectiveness of MICP for successful elimination of Pb^2+^ and Hg^2+^ was examined. The parametric changes of media chemistry in remediation trials were demonstrated. Experimental results denoted the sufficient removal of Pb^2+^ (95.2 and 91.1%) and Hg^2+^ (92 and 88.3%) from initial 350 ppm following incubation at (144 and 168 hr.) and (168 and 186 hr.) under aerobic and anaerobic conditions, respectively. Mineralogical analysis including EDS, SEM and XRD confirmed the existence of calcite and cerussite in both Pb^2+^ remediated samples. However, HgO and Hg, conjugated with calcite, were observed in aerobic and anaerobic Hg^2+^ remediated samples, respectively; which emphasized the uptake of soluble toxic metals and their transformation to insoluble stable form entrapped in calcite lattice. Such metal-calcite deposits provided an advantage in limiting the leaching of metals from carbonate bound complex to the surrounding environment. Such can be considered as a good strategy for cost-effective, environmentally friendly and effective method for heavy metals bioremediation.

## Supplementary information

Supplementary information.

## Data Availability

All data generated or analyzed during this study are included in this article (and its Supplementary Information File).
