# Supplementary material for: Aerobic and anaerobic removal of lead and mercury via calcium carbonate precipitation mediated by statistically optimized nitrate reductases
Source: Sci Rep. 2020 Mar 4;10:4029. doi: 10.1038/s41598-020-60951-1 (PMC7055279; doi:10.1038/s41598-020-60951-1)
Supplement: Supplementary file 1 — Supplementary information. [file 41598_2020_60951_MOESM1_ESM.docx]

**Aerobic and anaerobic removal of lead and mercury via calcium carbonate precipitation mediated by statistically optimized nitrate reductases**

Marwa Eltarahony^1*^, Sahar Zaki^1^, Desouky Abd-El-Haleem^1^

^1^Environmental Biotechnology Department, Genetic Engineering and Biotechnology Research Institute (GEBRI), City of Scientific Research and Technological Applications (SRTA-City), 21934, New Borg El-Arab City, Alexandria, Egypt

* **Corresponding author.**

**E-mail:** [m_eltarahony@yahoo.com](mailto:m_eltarahony@yahoo.com)

[meltarahony@srtacity.sci.eg](mailto:meltarahony@srtacity.sci.eg)  **(**Marwa Eltarahony**)**

**(Supplementary Table S1):** Statistical analysis of Plackett–Burman design showing coefficient, *t-*test values, *P-*values and confidence level (%) for variables affecting NAP activity.

| **Predictor (Symbol)** | **Coef** | **SE Coef** | **T** | ***P*** | **Confidence level %** |
| --- | --- | --- | --- | --- | --- |
| **Constant** | 617.8 | 135.3 | 4.56 | 0.02 | 98 |
| **pH (X1)** | -88.83 | 14.81 | -6 | 0.009 | 99.1 |
| **Inoculum Size (X2)** | -11.79% | 1.975 | -5.97 | 0.009 | 99.1 |
| **FeCL_2_.4H_2_O (X3)** | 0.61965 | 0.07405 | 8.37 | 0.004 | 99.6 |
| **CuSO_4_.5H_2_O (X4)** | 0.1746 | 0.1646 | 1.06 | 0.367 | 63.3 |
| **MnCl_2_·4H_2_O (X5)** | -0.9739 | 0.3291 | -2.96 | 0.06 | 94 |
| **Sodium Citrate (X6)** | 50.409 | 7.405 | 6.81 | 0.006 | 99.4 |
| **K_2_HPO_4_ (X7)** | -15.27 | 9.873 | -1.55 | 0.22 | 78 |
| **MgSO_4_ (X8)** | 332.1 | 123.4 | 2.69 | 0.074 | 92.6 |
| **ZnSO_4_·7H_2_O (X9)** | -0.18414 | 0.07405 | -2.49 | 0.089 | 91.1 |
| **CoCl_2_·6H_2_O (X10)** | -0.0679 | 0.2468 | -0.28 | 0.801 | 19.9 |
| **KH_2_PO_4_ (X11)** | 8.313 | 9.873 | 0.84 | 0.462 | 53.8 |
| **Aeration (X12)** | 0.9106 | 0.2962 | 3.07 | 0.054 | 94.6 |
| **H_3_BO_4_ (X13)** | -0.0308 | 0.04557 | -0.68 | 0.548 | 45.2 |
| **Na₂MoO₄.2H₂O (X14)** | 2.8089 | 0.2962 | 9.48 | 0.002 | 99.8 |
| **NaNO_3_ (X15)** | 18.579 | 5.924 | 3.14 | 0.052 | 94.8 |
| **NaCL (X116)** | -39.51 | 14.81 | -2.67 | 0.076 | 92.4 |
| **S =** 66.2333 | **R^2^ =** 99.1% | **R^2^(adj) =** 94.3% | |  |  |

**(Supplementary Table S2):** Statistical analysis of Plackett–Burman design showing coefficient, *t-*test values, *P-*values and confidence level (%) for variables affecting NAR activity.

| **Term (symbol)** | **Coef** | **SE Coef** | **T** | ***P*** | **Confidence Level %** |
| --- | --- | --- | --- | --- | --- |
| **Constant** | 437.6 | 278.3 | 1.57 | 0.214 | 78.6 |
| **KH_2_PO_4_ (X1)** | 16.54 | 14.35 | 1.15 | 0.333 | 66.7 |
| **MgSO_4_ (X2)** | 244.6 | 143.5 | 1.7 | 0.187 | 81.3 |
| **NaCL (X3)** | 53.39 | 28.71 | 1.86 | 0.16 | 84 |
| **CoCl_2_·6H_2_O (X4)** | -1.0377 | 0.3588 | -2.89 | 0.063 | 93.7 |
| **NaNO_3_ (X5)** | 44.29 | 10.77 | 4.11 | 0.026 | 97.4 |
| **FeCL_2_.4H_2_O (X6)** | 0.8371 | 0.1077 | 7.78 | 0.004 | 99.6 |
| **CuSO_4_.5H_2_O (X7)** | 0.7612 | 0.2392 | 3.18 | 0.05 | 95 |
| **K_2_HPO_4_ (X8)** | -10.71 | 14.35 | -0.75 | 0.51 | 49 |
| **pH (X9)** | -114.91 | 28.71 | -4 | 0.028 | 97.2 |
| **ZnSO_4_·7H_2_O (X10)** | -0.3387 | 0.1077 | -3.15 | 0.051 | 94.9 |
| **Na₂MoO₄.2H₂O (X11)** | 1.4771 | 0.4306 | 3.43 | 0.042 | 95.8 |
| **MnCl_2_·4H_2_O (X12)** | 0.1957 | 0.4785 | 0.41 | 0.71 | 29 |
| **H_3_BO_4_ (X13)** | -0.16209 | 0.06835 | -2.37 | 0.098 | 90.2 |
| **Sodium citrate (X14)** | 57.35 | 10.77 | 5.33 | 0.013 | 98.7 |
| **Inoculum Size (X15)** | 23.96% | 10.77 | 2.23 | 0.112 | 88.8 |
| **Temperature (X16)** | 1.084 | 4.306 | 0.25 | 0.817 | 18.3 |
| **S** = 96.2893 | **R^2^ =** 98.37% | | | **R^2^ (adj) =** 89.67% | |

**(Supplementary Figure S3):** Normal probability plot of variables (A) and Pareto chart of independent factors (B) affecting on NAP activity of strain 10B


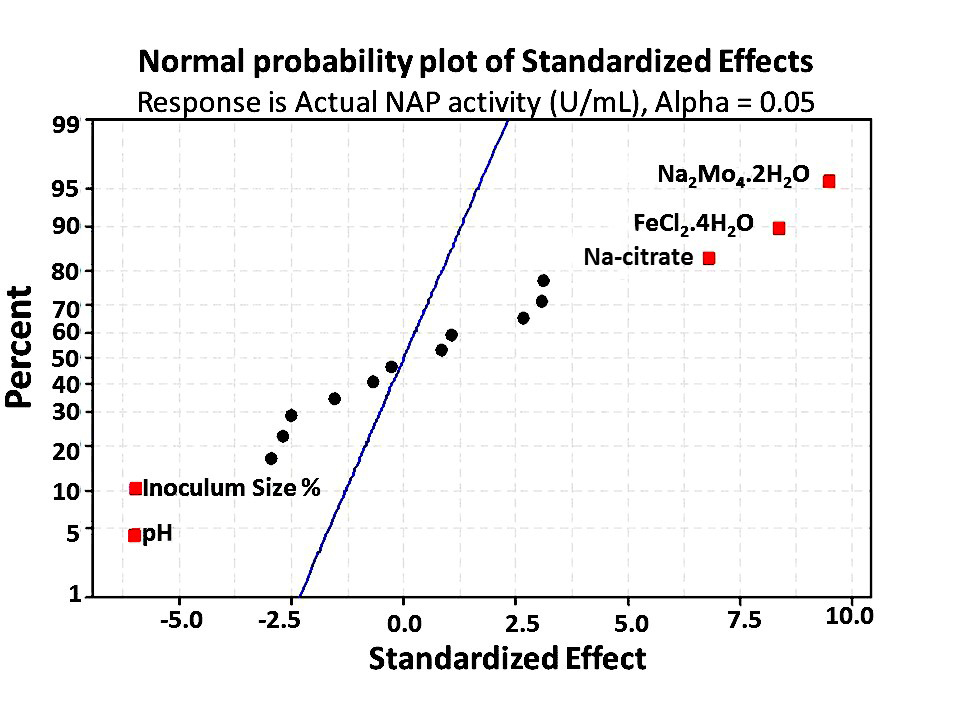


**A**


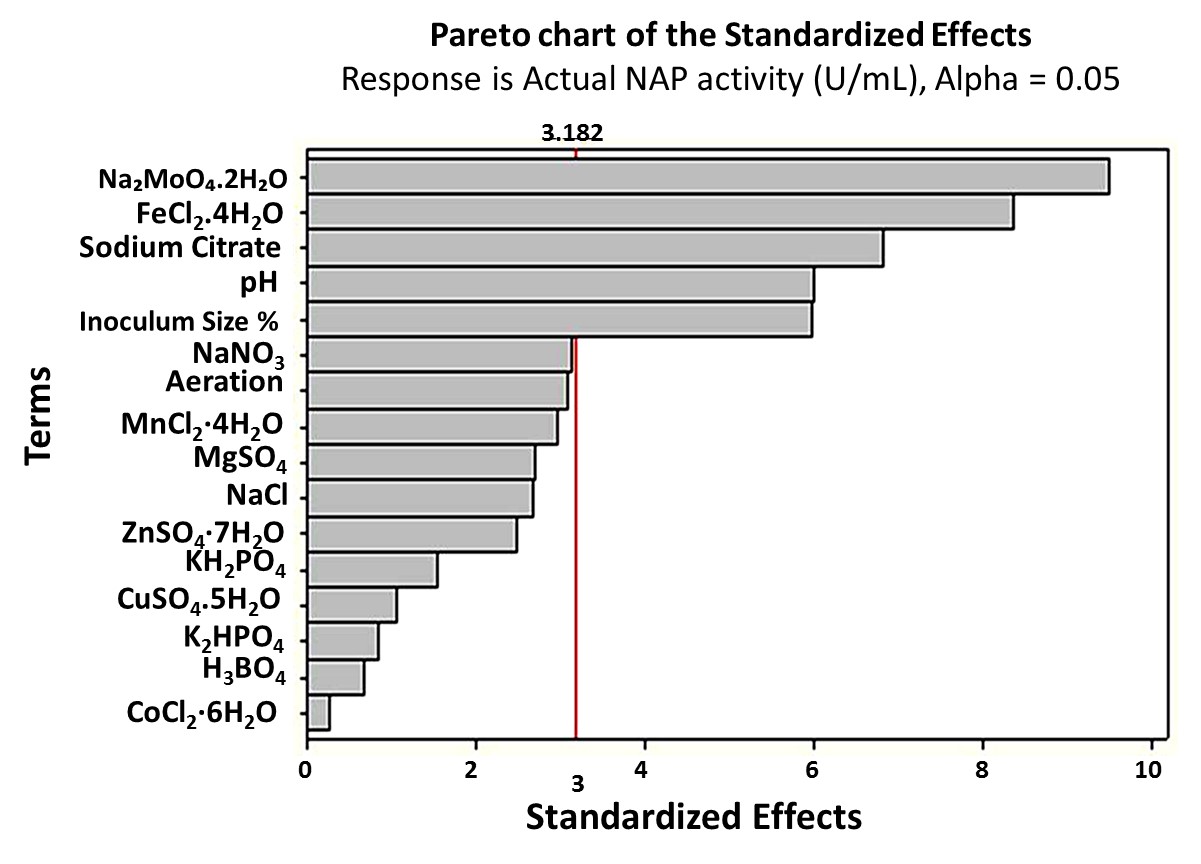


**B**

The Pareto chart which has been described as a useful tool for identifying the order of significance. As depicted, the factors Na_2_MoO_4_·2H_2_O, FeCl_2_·4H_2_O, sodium citrate, pH and inoculum size ranked the highest coefficient estimate, which were selected for CCD. For a 95% confidence level and three degrees of freedom, the 𝑡 value equals 3.18 and is shown in the plot as a vertical red line. This indicates the minimum statistically significant effect for 95% confidence level. So, the rest factors were not significant as their values not exceeding the t-value (red line).

**(Supplementary Figure S4):** Normal probability plot of variables (A) and Pareto chart of independent factors (B) affecting on NAR activity of strain 10B


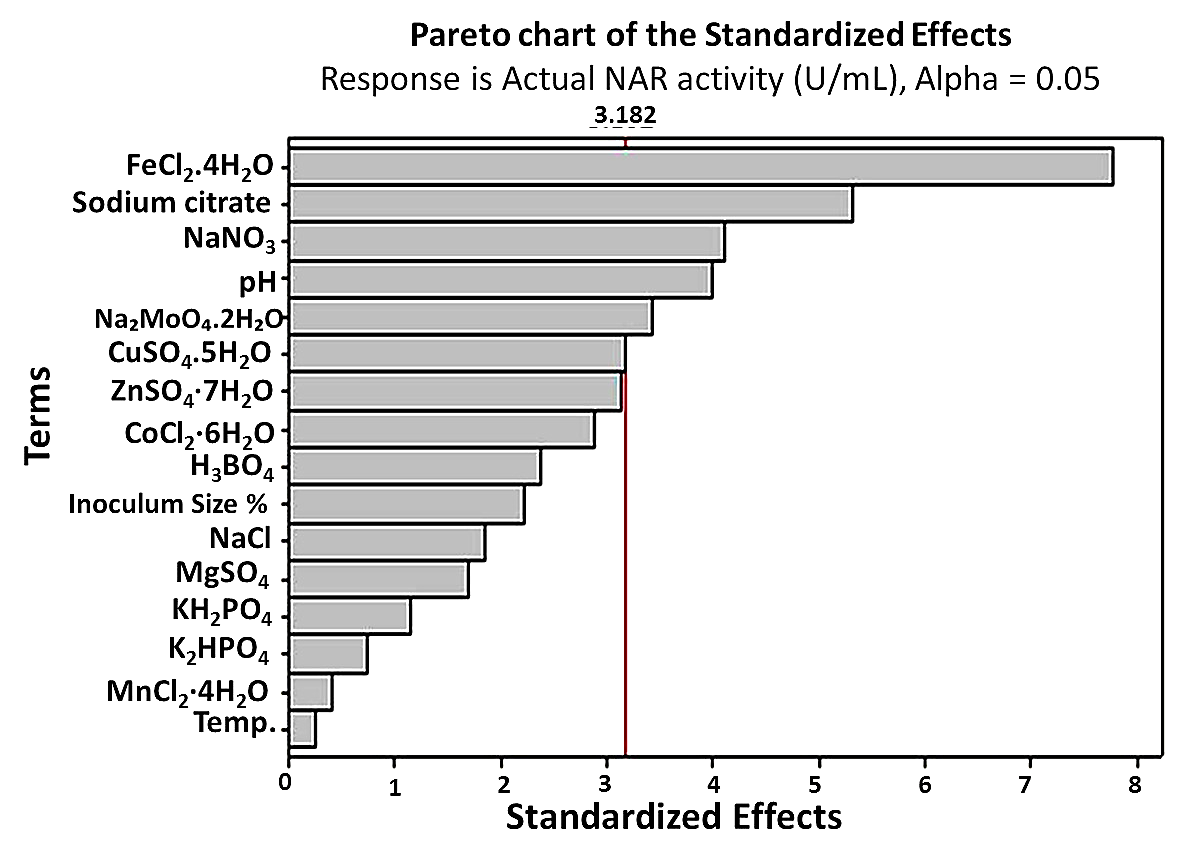


**B**


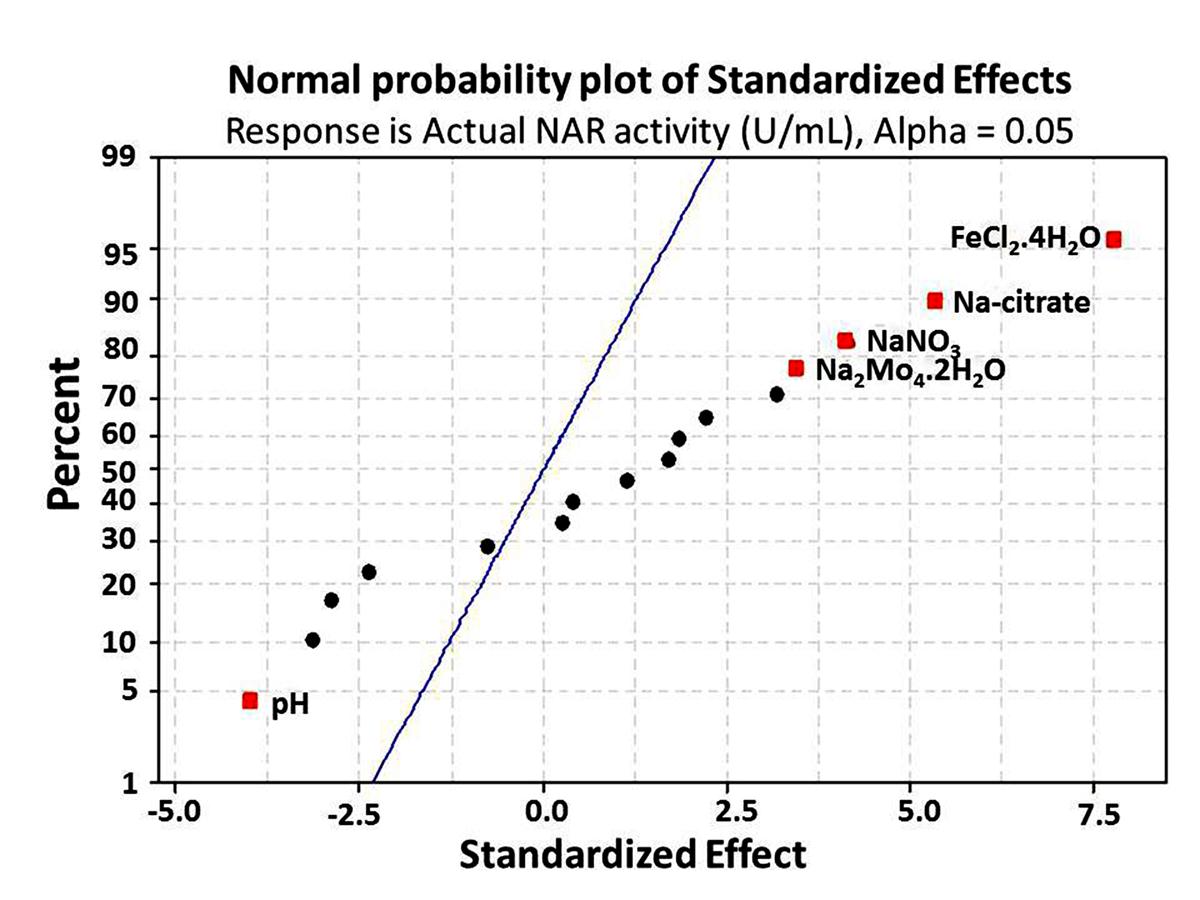


**A**

As shown, the factors FeCl_2_·4H_2_O, sodium citrate, NaNO_3_, pH and Na_2_MoO_4_·2H_2_O ranked the highest coefficient estimate and so were selected for further optimization. For a 95% confidence level and three degrees of freedom, the 𝑡 value equals 3.18 is shown in the plot as a vertical red line. This indicates the minimum statistically significant effect for 95% confidence level. So, the rest factors were not significant as their values not exceeding the t-value (red line).

**(Supplementary Table S5):** ANOVA for quadratic model of NAP/NAR activity of strain 10B

| **Source** | **Df** | | **Seq SS** | | **Adj SS** | | **Adj MS** | | ***F*** | | ***P*** | |
| --- | --- | --- | --- | --- | --- | --- | --- | --- | --- | --- | --- | --- |
|  | **NAP** | **NAR** | **NAP** | **NAR** | **NAP** | **NAR** | **NAP** | **NAR** | **NAP** | **NAR** | **NAP** | **NAR** |
| **Main effect** | 16 | 16 | 1453010 | 1678104 | 1453010 | 1678104 | 90813 | 104881 | 20.7 | 11.31 | 0.015 | 0.035 |
| **Residual Error** | 3 | 3 | 13161 | 27815 | 13161 | 27815 | 4387 | 9272 |  |  |  |  |
| **Total** | 19 | 19 | 1466170 | 1705919 |  |  |  |  |  |  |  |  |
